# Comparative efficacy of bio-selenium nanoparticles and sodium selenite on morpho-physiochemical attributes under normal and salt stress conditions, besides selenium detoxification pathways in *Brassica napus* L.

**DOI:** 10.1186/s12951-022-01370-4

**Published:** 2022-03-27

**Authors:** Ali Mahmoud El-Badri, Ahmed M. Hashem, Maria Batool, Ahmed Sherif, Elsayed Nishawy, Mohammed Ayaad, Hamada M. Hassan, Ibrahim M. Elrewainy, Jing Wang, Jie Kuai, Bo Wang, Shixue Zheng, Guangsheng Zhou

**Affiliations:** 1 MOA Key Laboratory of Crop Ecophysiology and Farming System in the Middle Reaches of the Yangtze River, College of Plant Science & Technology, Huazhong Agricultural University, Wuhan, 430070 People’s Republic of China; 2 Field Crops Research Institute, Agricultural Research Center (ARC), Giza, 12619 Egypt; 3 Biotechnology Department, Faculty of Agriculture, Al-Azhar University, Cairo, 11651 Egypt; 4 Desert Research Center, Genetics Resource Department, Egyptian Deserts Gene Bank, Cairo, 11735 Egypt; 5 Plant Research Department, Nuclear Research Center, Atomic Energy Authority, Abo Zaabal, Cairo, 13795 Egypt; 6 State Key Laboratory of Agricultural Microbiology, College of Life Science and Technology, Huazhong Agricultural University, Wuhan, 430070 People’s Republic of China

**Keywords:** Bio-selenium nanoparticles, Sodium selenite, Selenium detoxification, Salt stress, *Brassica napus* L.

## Abstract

Selenium nanoparticles (SeNPs) have attracted considerable attention globally due to their significant potential for alleviating abiotic stresses in plants. Accordingly, further research has been conducted to develop nanoparticles using chemical ways. However, our knowledge about the potential benefit or phytotoxicity of bioSeNPs in rapeseed is still unclear. Herein, we investigated the effect of bioSeNPs on growth and physiochemical attributes, and selenium detoxification pathways compared to sodium selenite (Se (IV)) during the early seedling stage under normal and salt stress conditions. Our findings showed that the range between optimal and toxic levels of bioSeNPs was wider than Se (IV), which increased the plant’s ability to reduce salinity-induced oxidative stress. BioSeNPs improved the phenotypic characteristics of rapeseed seedlings without the sign of toxicity, markedly elevated germination, growth, photosynthetic efficiency and osmolyte accumulation versus Se (IV) under normal and salt stress conditions. In addition to modulation of Na^+^ and K^+^ uptake, bioSeNPs minimized the ROS level and MDA content by activating the antioxidant enzymes engaged in ROS detoxification by regulating these enzyme-related genes expression patterns. Importantly, the main effect of bioSeNPs and Se (IV) on plant growth appeared to be correlated with the change in the expression levels of Se-related genes. Our qRT-PCR results revealed that the genes involved in Se detoxification in root tissue were upregulated upon Se (IV) treated seedlings compared to NPs, indicating that bioSeNPs have a slightly toxic effect under higher concentrations. Furthermore, bioSeNPs might improve lateral root production by increasing the expression level of *LBD16*. Taken together, transamination and selenation were more functional methods of Se detoxification and proposed different degradation pathways that synthesized malformed or deformed selenoproteins, which provided essential mechanisms to increase Se tolerance at higher concentrations in rapeseed seedlings. Current findings could add more knowledge regarding the mechanisms underlying bioSeNPs induced plant growth.

**Graphical Abstract:**

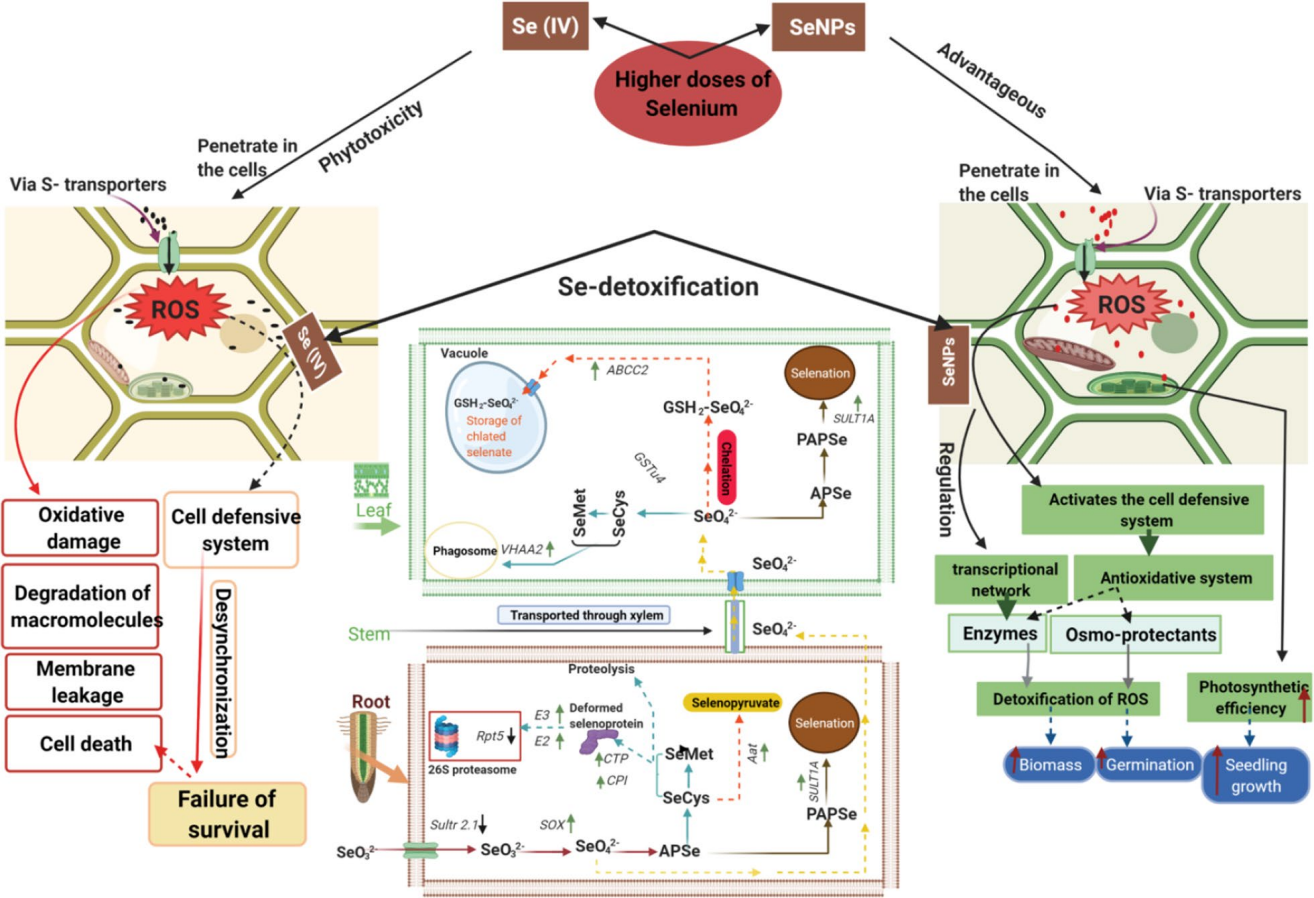

**Supplementary Information:**

The online version contains supplementary material available at 10.1186/s12951-022-01370-4.

## Introduction

Selenium is a fundamental and beneficial element for different organisms at low exposure levels and toxic at high concentrations with a narrow range between deficiency and excess [1]. Under lower doses, Se enhances the plant growth parameters by alleviating abiotic stresses effects such as organic and inorganic pollutants, salt stress, elevated temperature and water deficiency [2]. Selenium can enter directly or indirectly into the food chain mainly via plants; therefore, studying the impacts and fate of Se is significant for living organisms. Also, Se participates in the vital biological processes by synthesizing into selenoenzymes, which can improve the plant antioxidant activity, scavenge free radicals and protect the cell membrane [3]. On the contrary, Se with higher doses acts as a pro-oxidant, reducing yields and inducing metabolic disturbances [4]. The effects of Se in different tissues are dependent on the exposure doses and type of Se [5]. Furthermore, Se uses the same pathway as sulfur (S) in the plant, and it’s transported into plant tissues using S transporters and assimilated to selenocysteine (SeCys) or selenomethionine (SeMet); besides, Se might be methylated and converted into a non-toxic form [2, 6, 7] (Additional file 1: Fig. S1).

In the last decade, the application of several metal-based NPs such as ZnONPs [8], FeNPs and ZnNPs [9], TiO_2_NPs [10], CuNPs [11] and AgNPs [12] reduced the harmful effects of respective metal elements in agriculture. It also reduced the cost of fertilizers and pesticides, improved the efficiency of chemical materials, enhanced the absorbance, transportation and transformation of these minerals from the soil into the plant; hence it improved the productivity of plants and bio-controlling [13, 14]. Nanotechnology has the potential to revolutionize agriculture and play an important role in food and crop production [12, 15]. Compared to bulk materials, nanoparticles possess better physicochemical properties at optimum concentrations [16, 17].

Nanotechnology can assist the synthesis of different antioxidative compounds based upon their redox abilities using minerals, among them is Se, which was considered to rely on its red-ox abilities, owing to its various oxidation forms (^+^6, ^+^4, ^+^2, 0, ^−^1, ^−^2), besides that it also has a complicated antioxidative efficiency [18]. Selenium nanoparticles (SeNPs) were synthesized using biotic or abiotic pathways and widely occurred in the ambient, particularly in the heavy metallic mining regions [18]. The form of SeNPs is reported as novel compounds with lower toxicity compared with other seleno-species; additionally, it has better antioxidant properties due to the zero-oxidation state [19]. The significant effects of SeNPs on various species alter according to their different growth stages and exposure periods depending on SeNPs physiochemical composition [20]. SeNPs treatment reinforced plant growth in mustard, tomato and tobacco [13–15], improved POD activity, and reduced MDA content through ROS suppression and inhibiting free radical activity [21]. SeNPs play a promising role in various essential metabolic and physiochemical processes, thereby improving plant development [22]. Under the SeNPs application, no impact was found on the photosynthetic efficiency, demonstrated by its limited permeation in the leave cells [23]. The stimulatory effects of SeNPs were due to their slow uptake but rapid oxidation to selenite (become organic forms SeCys and SeMet) inside the plant [24].

Salinity is one of the major abiotic environmental stresses affecting plant crops, which involved 7% of rainfed and 30% worldwide irrigated agriculture, finally leading to a 65% loss of crop production [25]. Salinity affects plant growth due to the toxicity of Na^+^ and decreases the uptake of essential nutrients, including calcium (Ca^2^^+^) and potassium (K^+^), which disrupt cellular structures and restrict growth [26]. A higher salt level causes both osmotic and ionic stresses, damaging the photosynthetic apparatus and physiological processes such as closing the stomata and reducing the leaf expansion [27]. Additionally, salinity-induced osmotic stress is often accompanied by secondary stresses, such as oxidative stress that is harmful to plant cells due to excessive ROS, osmotic imbalance and water deficiency, which result in ion toxicity in stressed plants [28].

The current findings visualized the beneficial role of SeNPs by improving photosynthesis and antioxidative responses with optimal supplementation during the early growth stages [29]. We need more research to systematically comprehend the different pathways and interactions between selenium and plants, where studies on toxic effects and their conduct are still finite. Se’s hyperaccumulation ability in *Brassicaceae*, *Asteraceae* and *Fabaceae* has been reported [2]. Several studies have investigated *Brassicaceae,* especially *B. napus,* as it is the secondary accumulator model of Se with no signs of toxicity up to 100–1000 mg Se Kg^−1^ DW [2]. *Brassica napus* L. is one of the world’s most important sources of high-quality vegetable oils with vegetable protein diets for livestock and human nutrition [30].

In this study, we scrutinize the Se-prompted dual impact on the physiochemical and molecular mechanisms of rapeseed. Hence*,* we applied two forms of Se [Na_2_SeO_3_ (Se (IV)) and bioSeNPs] in the culture solution to estimate the phytotoxicity of higher selenium on seed germination and early seedling growth through morpho-physiochemical properties under normal and salt stress conditions. Besides, we investigated the selenium detoxification pathways in rapeseed seedlings under higher doses of selenium during the early seedling stage in *B. napus*.

## Materials and methods

### Preparation, purification and characterization of bio selenium nanoparticles (bioSeNPs)

A 1% culture of *Comamonas testosteroni* S44 was inoculated in the LB media (LB, Sigma) and incubated at 37 °C for 12 h, 10 mM sodium selenite [(Na_2_Se_2_O_3_) (Se (IV)] was added to the culture media and incubated at 28 °C for further 72 h. The appearance of red color indicating the production of elemental selenium. Precipitated cells were washed 2–3 times with ddH_2_O (18.2 MΩ·cm) and lysed by ultrasonication followed by centrifugation at 12,000 rpm for 5 min at room temperature. The pellets were resuspended and centrifuged with 80% (w/v) sucrose to remove the biomass (Additional file 1: Fig. S2). After that, the pellets were washed twice with ddH_2_O to purify the bioSeNPs and kept at − 20 °C [31, 32].

To understand the characterization of bioSeNPs, the size distribution (DLS) and zeta potential were determined using zetasizer 2000 (UK). BioSeNPs were prepared for fourier transform infrared (FTIR) spectroscopy analysis. The sample was mixed with spectroscopic grade potassium bromide (KBr, dried for 24 h at 60 °C) in a ratio of 1:100, and the spectrum was recorded in the range of 400–4000 wavenumber (cm^−1^) on the FTIR spectrometer, Spectrum 100 (Perkin Elmer, USA) in the diffuse reflectance mode at a resolution of 4 cm^−1^ in KBr pellets.

Structural properties of bioSeNPs were measured by scanning electron microscopy (SEM) using a Philips JSM 6390 model (USA) electron microscope and transmission electron microscopy (TEM) (JEM-2100F, JEOL Inc.) at an accelerating voltage of 15 kV and 200 kV, respectively.

### Plant material and treatment conditions

The mature seeds of rapeseed cultivar Yangyou 9 [Chinese rapeseed cultivar (扬油9号) collected from Jiangsu Lixiahe Agricultural Research Institute] were sterilized with 5% NaClO for five minutes then washed by ddH_2_O 4–5 times. Seeds of uniform size were selected to minimize errors in seed germination and seedling vigor. To investigate the effect of bioSeNPs and Se (IV) on *B. napus* under standard and salt stress conditions, 60 sterilized seeds were placed on filter paper in the germination boxes (15 × 10 × 5 cm), containing 15 mL of different solutions, ddH_2_O as a control and 50, 100 and 150 µmol L^−1^ Se (IV) or bioSeNPs (standard conditions), ddH_2_O, 150 and 200 mM NaCl as a control, 50, 100 and 150 µmol L^−1^ Se (IV) or bioSeNPs combined with 150 and 200 mM NaCl (salt stress conditions). Three replicates of seeds were kept according to a randomized block design in a growth chamber with optimal conditions (day/night temperature at 25 ± 1/20 ± 1 °C) with 12 h light (13,000 lx) and 12 h dark (HP250GS-C, Ruihua Instrument and Equipment Co., Ltd., Wuhan, China) according to [33]. Germinated seeds were counted daily, starting from the first day of cultivation to the seventh day; seeds were considered germinated when the primary root was at least 2 mm long. All samples were collected and stored at − 80 °C to determine physiochemical attributes.

### Morphological characters

The final germination percentage (FG%), germination rate (GR), vigor index I (VI (I)) and vigor index II (VI (II)) were measured according to the equations reported by [12, 34].

After seven days of treatments, plants were harvested and separated into shoots and roots to measure lengths and fresh weight then dried to a constant weight at 80 °C for dry weight measurements [35].

### Measurement of photosynthetic pigments

The contents of total chlorophyll and carotenoids in fresh samples were determined according to the formulae suggested by [36, 37].

### Total soluble protein, total soluble sugar, malondialdehyde (MDA) and proline contents

Total soluble protein content was measured following [38] with BSA as a standard protein. At the same time, the total soluble sugar content was estimated according to [39]. MDA content was measured using thiobarbituric acid (TBA), as mentioned by [40]. The absorbance of proline content was measured at 520 nm using toluene as a blank, according to [41].

### Determination of H_2_O_2_ and O_2_^−^ by NBT and DAB staining

3,3′-Diaminobenzidine (DAB, 1 mg mL^−1^) and Nitrotetrazolium blue (NBT, 1 mg mL^−1^) staining were used to detect H_2_O_2_ and O_2_^−^ levels, respectively, in leaves and roots. DAB and NBT staining were performed as described by [42, 43].

### RNA extraction, cDNA synthesis and quantitative RT-PCR

Seven-day after germination of *B. napus,* shoots and roots were collected and immediately frozen in liquid nitrogen for RNA extraction. Total RNA extraction was performed using TransZol Up reagent (TransGen, Beijing, China). Concentration and quality of RNA were determined with a Nanodrop 2000 Spectrophotometer (Thermo Fisher Scientific). cDNA was synthesized from 1 µg of RNA using TransScript One-Step gDNA Removal and cDNA Synthesis SuperMix kit (TransGen, Beijing, China) following the manufacturer’s instructions. cDNA was diluted 1:10 for quantitative real‐time PCR (qRT-PCR).

Quantitative real-time PCR analyses were performed using TransStart Tip Green qPCR SuperMix (TransGen, Beijing, China) with Roche LightCycler 480 thermal cycler instrument, 384-well (Roche). Relative expression values were calculated using the 2^−ΔΔCt^ method according to [44, 45], *β-ACTIN* was used as a reference gene, primers are listed in Additional file 1: Table S1. Three biological replicates were performed for each sample, and three technical replicates represented each biological replicate.

### Statistical analysis

The data for each variable were subjected to analysis of variance. The significance of differences between the control and the treatment mean values were determined by the Duncan’s Multiple Range Test (DMRT) at *P* < 0.05 significance level. The graphical presentation was carried out using GraphPad prism (V8.0.1) and RStudio software. The values presented are the means of three independent experiments.

## Results

### BioSeNPs characterization

**Fig. 1:**
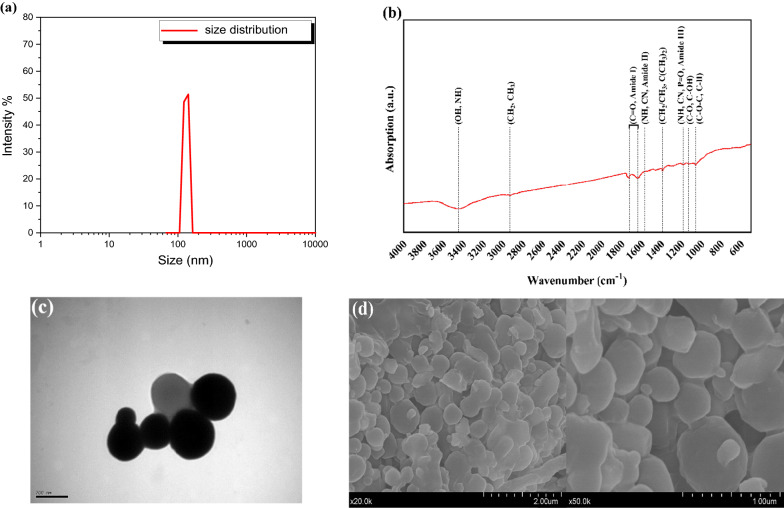
Characterization of bioSeNPs **a** size distribution; **b** fourier transform infrared spectrum (FTIR); **c** transmission electron microscopic image (TEM) and **d** scanning electron microscopic image (SEM) of SeNPs produced in *C. testosteroni* S44

Dynamic Light Scattering (DLS) analysis showed that the size of bioSeNPs was ranged from 120 to 260 nm with an average size of 167 nm (Fig. 1a). However, a few bioSeNPs displayed an extremely big size. The possible explanation could be that a high concentration of bioSeNPs formed large aggregates; meanwhile, zeta potential analysis indicates that bioSeNPs displayed a negative potential of − 32.4 ± 2 mV in ddH_2_O.

Fourier transform infrared (FTIR) spectroscopic analysis of bioSeNPs ranged from 400 to 4000 cm^−1^ (Fig. 1b). The FTIR spectrum of NPs exhibited peaks at 3446 cm^−1^ attributed to -OH and -NH stretching of protein, carbohydrates and lipids, and 2926 cm^−1^ can be ascribed to CH_2_ and CH_3_ stretching from lipids and proteins. Additionally, the strongest and sharp features at 1723 and 1638 cm^−1^ confirmed C=O stretching vibration present in proteins (amide I). The other band observed at 1567 cm^−1^ corresponded to N–H and C–N stretching vibrations, illustrating the presence of peptide bonds in different protein conformations (amide II). A sharp peak at 1385 cm^−1^ indicated the presence of CH_2_/CH_3_ and C(CH_3_)_2_ stretching mainly in proteins and lipids. The C–N stretching and N–H bending vibrations were also observed at 1181 cm^−1^ (amide III), which reflected the presence of the ν_asym_ PO_2_^−^ in nucleic acid and phospholipids on bioSeNPs. On the other side, small features of bioSeNPs at 1055 cm^−1^ (C–O–C and C–H) and 1128 cm^−1^ (C–O, C–OH) characterized to carbohydrates (Fig. 1b), these assignments are based on earlier research [31, 46, 47]. Conclusively, FTIR spectra confirmed the presence of various capping biomacromolecules (proteins, carbohydrates and lipids) at the SeNPs surface.

The morphology of biosynthesized SeNPs was visualized by transmission electronic microscopy (TEM) and scanning electronic microscopy (SEM) are shown in (Fig. 1c, d); bioSeNPs have a spherical architecture with an average diameter size ranging from 120 to 260 nm (TEM), and from 175 to 400 nm (SEM). Moreover, it was reported that the size of bioSeNPs was ranged from 120 to 300 nm [48].

### BioSeNPs enhanced seed germination and seedling growth

**Fig. 2:**
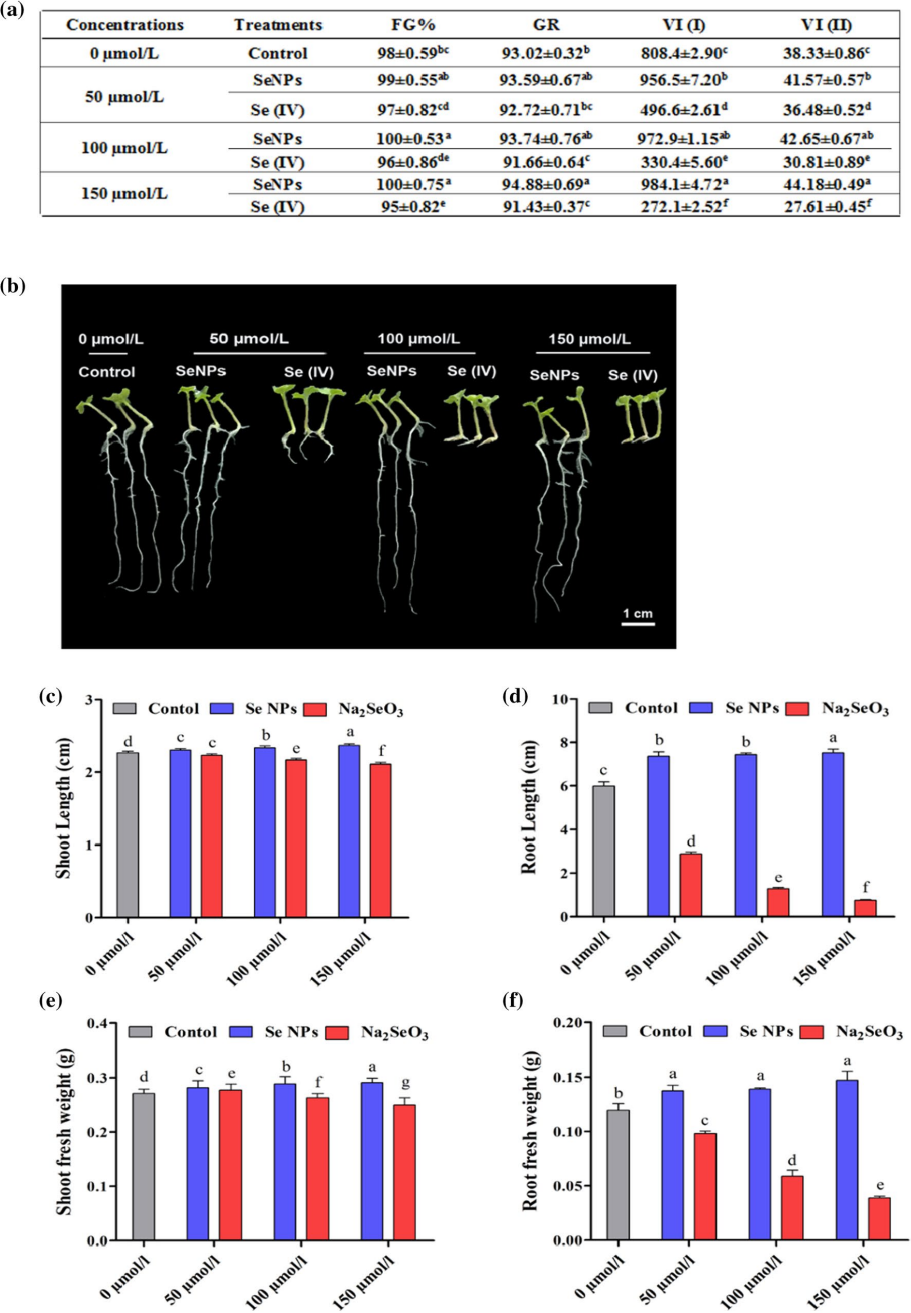
Effect of two selenium forms (SeNPs and Se (IV)) on rapeseed seedlings (Yangyou 9) during the early seedling stage. **a** impacts of selenium treatments on the germination characteristics (FG%: final germination percentage, GR: germination rate, VI (I): vigor index I and VI (II): vigor index II). SeNPs stimulated seedling growth. **b** Different concentrations of SeNPs and Se (IV) (0, 50, 100 and 150 µmol/L) differentially affected rapeseed seedlings phenotype. Scale bar = 1 cm. **c**–**f** Impact of selenium treatments on **c** shoot length (cm); **d** root length (cm); **e** shoot fresh weight (g) and **f** root fresh weight (g) under different concentration of SeNPs and Na_2_SeO_3_ (0, 50, 100 and 150 µmol/L) during the early seedling stage. Bars represent ± SE of three replicates. The different letters indicate significant differences at *P* < 0.05 using Duncan's multiple range tests

We exogenously applied different concentrations of bioSeNPs and Se (IV) (0, 50, 100 and 150 µmol L^−1^) to investigate the effect of Se (higher doses) on seed germination and seedling growth of *B. napus*. Our results demonstrated that the nano-solutions treatment accelerated the seed germination compared to sodium selenite and control. The final germination percentage (FG%) was significantly increased by 1.06, 1.30 and 1.27% under 50, 100 and 150 µmol L^−1^ doses of bioSeNPs versus control, respectively, while decreased by 0.86, 2.46 and 2.90% under 50, 100 and 150 µmol L^−1^ doses of Se (IV), respectively, compared to control (Fig. 2a). Interestingly, treated seeds with bioSeNPs, especially 150 µmol L^−1^, recorded the highest value of 94.88, 984.1 and 44.18% for germination rate (GR), vigor index I (VI (I)) and vigor index II (VI (II)), respectively, when compared to those of Na_2_SeO_3_ and control (Fig. 2a).

Furthermore, we investigated the effect of bioSeNPs on the phenotype of *B. napus* seedlings. We observed that after 7-days of exposure to bioSeNPs, plant growth was stimulated with no sign of toxicity, while the sodium selenite (Se (IV)) treatment showed wilt and stunted shoot and root growth compared to control (Fig. 2b).

Our results showed that 150 µmol L^−1^ bioSeNPs increased the shoot and root length by 8.47 and 24.74%, respectively, whereas Na_2_SeO_3_ (150 µmol L^−1^) decreased shoot and root length by 7.85 and 67.47% versus control, respectively (Fig. 2c, d). As compared to control, shoot fresh weight was significantly increased by 1.59, 3.10 and 4.49% under 50, 100 and 150 µmol L^−1^ of bioSeNPs, respectively, while it was decreased by 1.47, 4.32 and 6.89% under 50, 100 and 150 µmol L^−1^ of Na_2_SeO_3_, respectively (Fig. 2e). Besides, nano-treatments also increased the root fresh weight by 22.8% (50 µmol L^−1^), 24.24% (100 µmol L^−1^) and 25.5% (150 µmol L^−1^) versus control with the non-significant difference among the NPs concentrations. On the other side, Na_2_SeO_3_ reduced the root fresh weight by 51.87, 78.45 and 87.62% under 50, 100 and 150 µmol L^−1^, respectively, versus control (Fig. 2f). Additionally, Na_2_SeO_3_ treatments increased shoot dry weight by 29.56, 25.57 and 26.94%, while decreased root dry weight by 40.68, 63.52 and 71.42% under 50, 100 and 150 µmol L^−1^ versus control, respectively. In contrast, bioSeNPs have a non-significant effect on the shoot and root dry weight compared to relative control (Additional file 1: Fig. S3a, b).

### Twofold effects of two Se forms on photosynthetic pigments, osmoprotectants, MDA and proline contents

**Fig. 3:**
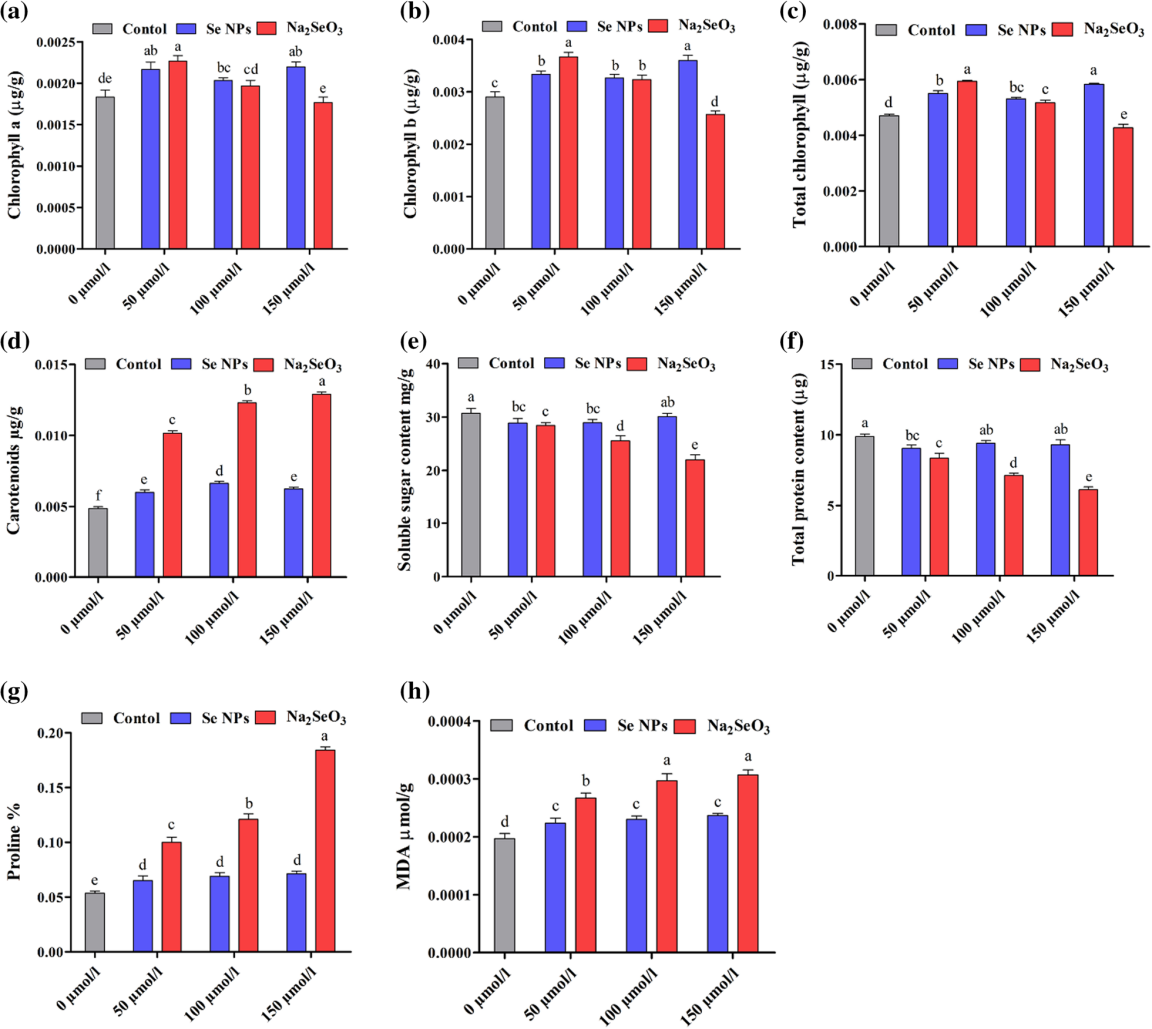
Effect of different doses of SeNPs and Na_2_SeO_3_ (0, 50, 100 and 150 µmol/L) on **a** chlorophyll a (µg/g); **b** chlorophyll b (µg/g); **c** total chlorophyll (µg/g); **d** carotenoids (µg/g); **e** total soluble sugar (mg/g); **f** total protein (µg); **g** proline % and **h** MDA (µmol/g) contents on rapeseed seedlings. Bars represent ± SE of three replicates. The different letters indicate significant differences at *P* < 0.05 using Duncan's multiple range tests

To investigate the effect of bioSeNPs and Na_2_SeO_3_ on photosynthesis, we analyzed the contents of the photosynthetic pigments upon Se treatments (Fig. 3a–d). Chlorophyll content was affected by Se application in both forms, especially on the higher concentration of Se (150 µmol L^−1^), which increased chlorophyll a, chlorophyll b and total chlorophyll contents by 21.83, 24.85 and 24.04% (bioSeNPs), while declining by 5.22, 11.14 and 8.19% (Na_2_SeO_3_), respectively, compared to the control (Fig. 3a–c). Moreover, carotenoids content dramatically increased with increasing the Se concentrations by 108.7, 151.3 and 164.6% (Na_2_SeO_3_)_,_ and by 23.13, 35.97 and 27.45% (bioSeNPs), under 50, 100 and 150 µmol L^−1^ versus control, respectively (Fig. 3d).

Our investigation demonstrated that the total soluble sugars (TSS) content showed a decreased trend with increasing Se concentrations. BioSeNPs decreased the TSS content by 6.01, 5.85 and 2.11%, and a large reduction was observed when Na_2_SeO_3_ was applied by 7.48, 16.89 and 28.48% under 50, 100 and 150 µmol L^−1^ versus control, respectively (Fig. 3e). Our results indicated a negative effect of Se on total soluble protein content (TSP) at the tested concentrations, which decreased by 8.48, 4.81 and 5.86% (bioSeNPs), 15.52, 27.81 and 38.05% (Na_2_SeO_3_) under 50, 100 and 150 µmol L^−1^ versus control, respectively (Fig. 3f).

The data (Fig. 3g) showed an increase in proline content corresponding to Se concentrations. The proline content showed significant increased by 21.60, 29.23 and 33.14% (bioSeNPs), 86.40, 125.69 and 243.38% (Na_2_SeO_3_) under 50, 100 and 150 µmol L^−1^, respectively, over the control. The same trend was observed for lipid peroxidation content; results showed significant differences in MDA among tested Se treatments. The high Se doses (150 µmol L^−1^) increased MDA content by 23.07 and 57.94% for bioSeNPs and Na_2_SeO_3_, respectively (Fig. 3h). Besides, bioSeNPs treatments showed lower MDA content than the Na_2_SeO_3_ treatments.

### Assessment of ROS accumulation and antioxidant enzyme genes expression under Se treatment

**Fig. 4:**
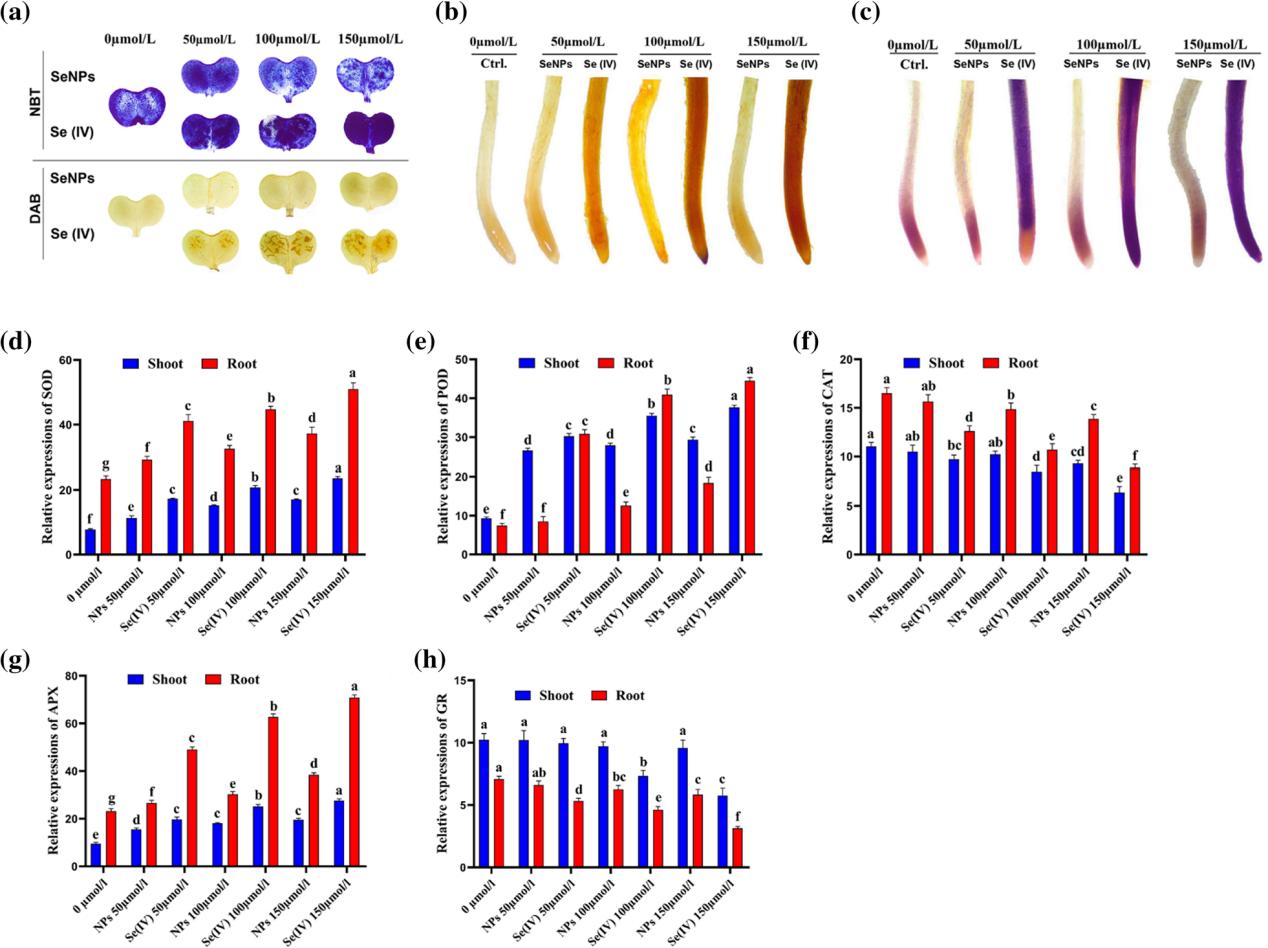
SeNPs modulated ROS accumulation and antioxidant enzymes related genes in rapeseed seedlings. **a** Nitro blue tetrazolium (NBT) and 3, 3-diaminobenzidine (DAB) staining accumulation under different doses of SeNPs and Se (IV) (0, 50, 100 and 150 µmol/L) on Yangyou 9 leaves; **b** roots stained with DAB; **c** roots stained with NBT. **d**–**h** The transcription levels of **d** superoxidase (SOD); **e** peroxidase (POD); **f** catalase (CAT); **g** ascorbate peroxidase (APX) and **h** glutathione reductase (GR) related genes in rapeseed shoot and root tissues. Bars represent ± SE of three replicates. The different letters indicate significant differences at *P* < 0.05 using Duncan's multiple range tests

After seven days of Se treatments, we investigated the accumulation of H_2_O_2_ and O_2_^•–^ in leaves and roots in rapeseed using nitro blue tetrazolium (NBT) and 3,3-diaminobenzidine (DAB), respectively. Results for DAB staining showed that Se (IV) treatments resulted in a darker brown color staining in leaves and roots than nano-treated, which elucidates the greater production and accumulation of excessive H_2_O_2_ in Se (IV) as compared to bioSeNPs at the same concentrations. Furthermore, the dark blue color of NBT staining represented a higher accumulation of O_2_^•–^ under Se (IV); meanwhile, the bioSeNPs treated seedling did not show an obvious effect on H_2_O_2_ and O_2_^•–^ accumulation in leaves and roots compared to control (Fig. 4a–c).

In this study, the transcript abundance of genes encoding antioxidant enzymes was differentially changed. After seven days of treatments, *B. napus* shoot and root tissues were harvested and used to determine the expression levels of *SOD, POD, APX, CAT* and *GR* genes under different doses of bioSeNPs or Se (IV). The relative mRNA levels of *SOD*, *POD* and *APX* were up-regulated by 204.0, 305.2 and 191.0% (shoots), 177.1, 500.1 and 205.2% (roots), and down-regulated by 42.22 and 43.83% (shoots), 46.30 and 55.66% (roots) with CAT and GR under 150 µmol L^−1^of Se (IV) versus control, respectively (Fig. 4d–h). On the other hand, under higher concentration (150 µmol L^−1^) of bioSeNPs, the expression pattern of these enzyme related genes was an increment in *SOD*, *POD* and *APX* by 119.0, 216.0 and 106.1% (shoots), 59.39, 148.00 and 65.73% (roots), respectively, while transcript level of *CAT* and *GR* genes was decreased by 15.84 and 6.40% (shoots), and 16.03 and 17.62% (roots), respectively, versus control (Fig. 4d–h).

### Impacts of Se application on growth during the early seedling stage

BioSeNPs boosted the germination parameters and growth traits under increased concentrations, with slight significance among the nano concentrations. In contrast, Se (IV) prominently inhibited the germination and growth traits and the maximum reduction in growth was expressly noted under the highest dose (150 µmol L^−1^). Moreover, total chlorophyll contents were affected by Se application, especially on the higher doses (150 µmol L^−1^) of bioSeNPs (increased) and Se (IV) (declined), while carotenoids content dramatically elevated by increasing the Se concentration versus control (Additional file 1: Fig. S4a–c). Similarly, all osmotic adjustment parameters were significantly influenced by maximum inhibition at 150 µmol L^−1^ compared to other treatments. The relative mRNA level of *SOD*, *POD*, and *APX* was up-regulated and down-regulated in *CAT* and *GR* under the highest Se (IV) doses in shoot and root tissues than relative control. The expression pattern of these enzyme-related genes was increased for *SOD*, *POD*, and *APX* in shoot and root tissues, while the transcript level of *CAT* and *GR* genes was decreased in shoots and roots under 150 µmol L^−1^ of bioSeNPs (Additional file 1: Fig. S4a–c).

### Principal component analysis of Se treated rapeseed plants

**Fig. 5:**
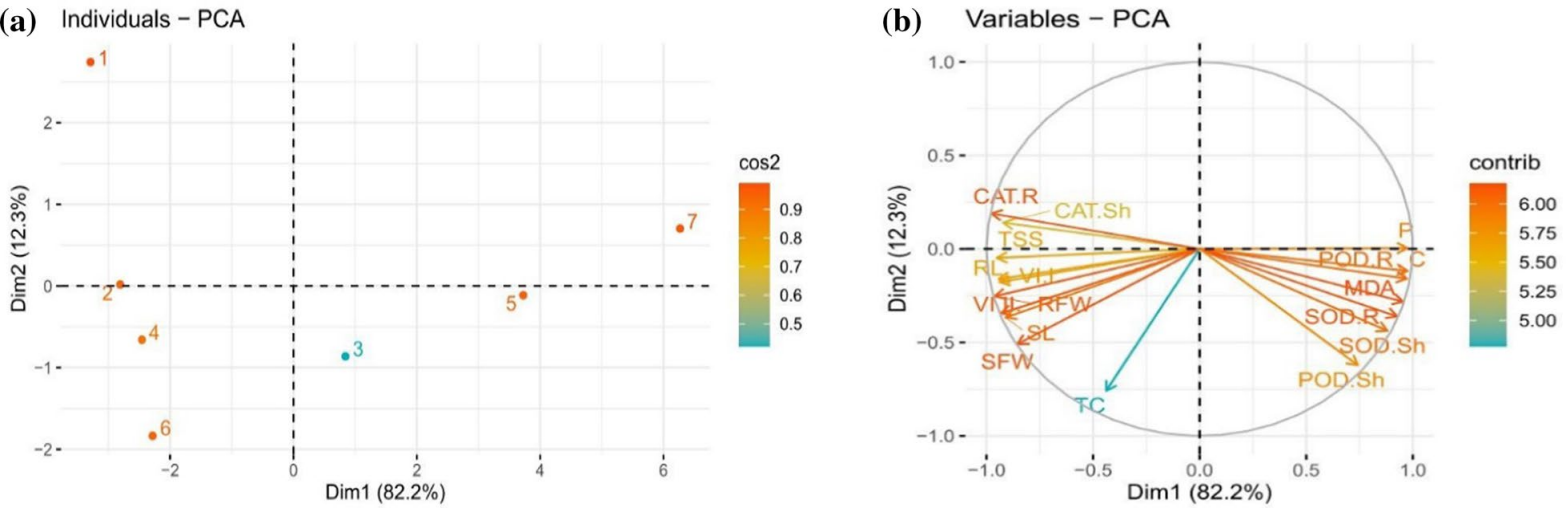
Principal component analysis (PCA) of SeNPs and Se (IV) treatments and variable trait relationship in rapeseed seedlings **a** PCA individual plots of Se treatments on rapeseed seedlings, and **b** PCA loading plots of PC1 and PC2 of the examined variable traits, and the circles indicate the most correlated variables. Score plot represents the separation of treatments as (1) Ck (0 µmol/L); (2) SeNPs 50 µmol/L; (3) Se (IV) 50 µmol/L; (4) SeNPs 100 µmol/L; (5) Se (IV) 100 µmol/L; (6) SeNPs 150 µmol/L and (7) Se (IV) 150 µmol/L. The tested variables included VI (I): vigor index I; VI (II): vigor index II; SL: shoot length; RL: root length; SFW: shoot fresh weight; RFW: root fresh weight; TC: total chlorophyll; C: carotenoids; TSS: total soluble sugar; MDA: malondialdehyde; P: proline content; SOD: superoxidase dismutase; POD: peroxidase; CAT: catalase in Sh: shoots, and R: roots; Dim1: dimension1; Dim2: dimension2; Cos2: squared cosine, and Contrib: contribution

All the 17 traits were loaded into two major principal components (PC1 and PC2), explaining 94.5% of the total variances. Most of the examined traits were discriminated by PC1, which was explained by the larger proportions of variances (82.2%), while the lower proportions of variances (12.3%) were indicated by PC2 (Fig. 5a, b). Doses distribution visualized a clear signal of elevated levels of bioSeNPs, which recorded significant positive effects on the studied characteristics of rapeseed, contrary to that of Se (IV), which recorded negative effects during the early seedling stage. Specifically, bioSeNPs were more displaced from the other treatments, indicating alleviation of the elemental toxicity on the seeds and enhanced germination and early seedling growth. In contrast, 150 µmol L^−1^ of Se (IV) was more displaced from the other treatments indicating increased elemental toxicity on the seeds and decreased germination and early seedling growth (Fig. 5a).

The loading plot classified the studied traits into three main groups depending on the two-dimensional plots. The CAT activity in shoot and root was positively correlated with Dim2, while MDA, proline and carotenoids contents were positively associated with Dim1. The variable MDA (lipid peroxidation) has positive loadings for PC1 (82.2% of the total variance), which confirmed that MDA content was strongly correlated (negatively) to germination and growth-related traits, including vigor index (I), vigor index (II), shoot, root fresh and dry weight, shoot, root length and total chlorophyll. On the other hand, it is revealed that almost all the growth traits are positively correlated to each other with varying degrees of relationship, while all these variables are negatively correlated to the oxidative biomarkers, including SOD and POD in shoots and roots (Fig. 5b).

### Impacts of Se treatments on the germination traits under two concentrations of NaCl

**Fig. 6:**
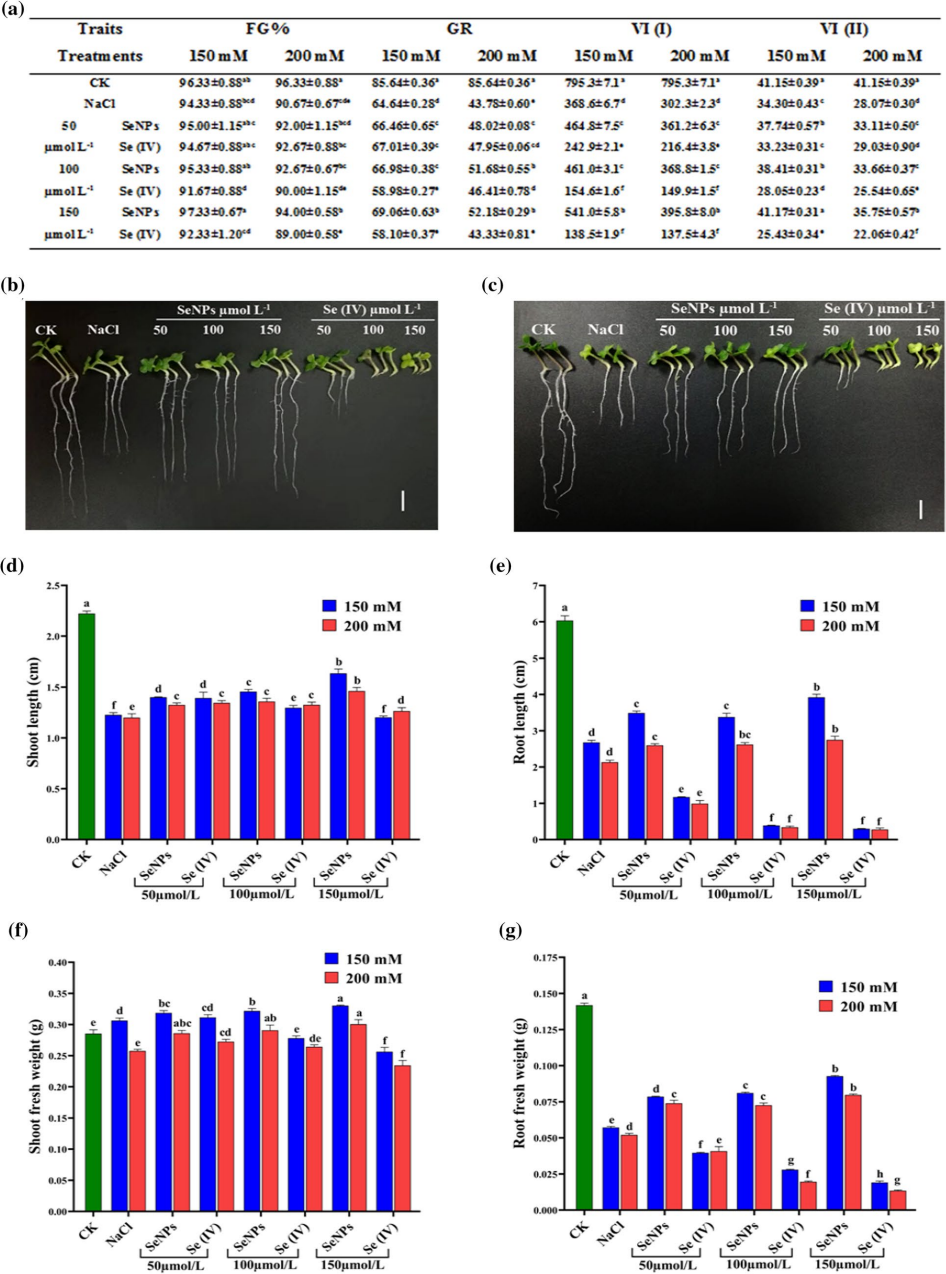
Nano-Se improves seedling growth under two different concentrations of NaCl. **a** Impact of selenium treatments on the germination characteristics [FG%: final germination percentage; GR: germination rate; VI (I): vigor index I and VI (II): vigor index II] under two concentrations of salt stress. **b** and **c** Seven-day-old seedlings phenotypes grown under two NaCl concentrations: **b** 150 mM NaCl and **c** 200 mM NaCl combined with different levels of SeNPs and Se (IV) (0, 50, 100 and 150 µmol/L). Scale bar = 1 cm. **d**–**g** Impact of selenium treatments on **d** shoot length; **e** root length; **f** shoot fresh weight and **g** root fresh weight under two concentrations of salt stress during the early seedling stage. Bars represent ± SE of three replicates. The different letters indicate significant differences at *P* < 0.05 using Duncan's multiple range tests

The findings of this study indicated that salt stress decreased the seeds germination by 2.08% (150 mM) and 6.21% (200 mM), which is more prominent under a higher stress level when comparing NaCl and CK. Whereas, supplementation of bioSeNPs increased the FG% by 3.18% (150 mM) and 3.68% (200 mM), while Se (IV) slightly decreased the FG% by 2.12% (150 mM) and 1.84% (200 mM) with 150 µmol L^−1^ versus the seedling treated with NaCl alone (Fig. 6a). Furthermore, bioSeNPs alleviated the salinity stress and improved the FG% compared to Se (IV). Increasing salt stress levels on stressed plants without Se treatments significantly decreased the GR by 24.53 and 48.88% under 150 and 200 mM NaCl versus CK. While, bioSeNPs application with 50, 100 and 150 µmol L^−1^ increased the GR by 2.81, 3.62 and 6.84% (150 mM), 9.69, 18.06 and 19.19% (200 mM) versus NaCl treated seedling alone. Besides this, Se (IV) strikingly 150 µmol L^−1^ decreased the GR by 10.11 and 1.03% under 150 and 200 mM, respectively, compared to salinized seedlings without Se treatment (Fig. 6a). Finally, Se (IV) decrement was lower than NaCl seedlings but higher than bioSeNPs treatments.

Furthermore, VI (I) and VI (II) are significantly decreased in NaCl treated seedlings by 53.65 and 16.35% (150 mM), 60.39 and 32.02% (200 mM), respectively, versus CK. However, bioSeNPs treatments with 50, 100 and 150 µmol L^−1^ increased the VI (I) by 26.07, 25.04 and 46.76% (150 mM), 19.50, 22.01 and 30.95% (200 mM), respectively, over NaCl treated seedlings alone. In contrast, Se (IV) decreased the VI (I) by 34.10, 58.06 and 62.42% (150 mM), 28.40, 50.41 and 54.51% (200 mM) under 50, 100 and 150 µmol L^−1^ doses, respectively, versus NaCl. Besides, VI (II) increased by 20.04% (150 mM) and 27.34% (200 mM) with bioSeNPs (150 µmol L^−1^), while Se (IV) decreased the VI (II) by 25.85% (150 mM) and 21.42% (200 mM) as compared to NaCl treated seedlings alone (Fig. 6a).

### Impacts of Se treatments on growth parameters under two concentrations of NaCl

The impact of two forms of Se treatments on rapeseeds under salt stress during the early seedling stage were evaluated by examining their effect on phenotypic appearance traits and vegetative biomass. The application of bioSeNPs had the most promising effect on promoting rapeseed seedling growth versus Se (IV) and NaCl under 150 and 200 mM of NaCl (Fig. 6b, c).

A considerable 44.79 and 46.87% decreases in the shoot length were noticed under 150 and 200 mM NaCl, respectively, versus CK (Fig. 6d). However, bioSeNPs treatments with 50, 100 and 150 µmol L^−1^ increased the shoot length by 14.10, 18.71 and 33.31% (150 mM), and 10.44, 13.22 and 21.81% (200 mM) over salinized seedlings alone (NaCl). Additionally, Se (IV) at 50 and 100 µmol L^−1^ elevated the shoot length by 13.46 and 5.67% (150 mM) and 12.11 and 10.52% (200 mM). Meanwhile, 150 µmol L^−1^ of Se (IV) decreased the shoot length by 1.99% under 150 mM but increased by 5.42% under 200 mM, compared to the seedlings treated with NaCl alone (Fig. 6d). With increasing the concentrations of bioSeNPs, the shoot length increment was higher than NaCl and Se (IV) treated seedlings.

The root length inhibition under salt stress was recorded as 55.57 and 65.05% reduction under 150 and 200 mM NaCl versus CK, respectively (Fig. 6e). However, bioSeNPs treatments increased the root length by 30.29, 26.10 and 46.33% (150 mM), and 21.85, 22.85 and 28.81% (200 mM), additionally, Se (IV) decreased the root length by 56.19, 85.47 and 88.91% (150 mM), and 53.54, 84.06 and 86.91% (200 mM) with 50, 100 and 150 µmol L^−1^ treatments, respectively, compared to the seedlings treated with NaCl alone, which suggested that Se (IV) highly negatively affected the seedlings comparing with NaCl and bioSeNPs treated seedlings (Fig. 6e).

The shoot fresh weight was increased under lower salt concentration 150 mM by 7.35%, while decreased by 9.73% under 200 mM comparison with CK. However, bioSeNPs treatments with 50, 100 and 150 µmol L^−1^ significantly increased shoot fresh weight by 4.03, 5.05 and 7.80% (150 mM), and 10.98, 12.86 and 16.70% (200 mM), respectively, over CK. Additionally, Se (IV) treatments increased the shoot fresh weight by 1.61% (150 mM) and 5.79% (200 mM) at 50 µmol L^−1^, while shoot fresh weight was decreased by 16.30% (150 mM) and 8.97% (200 mM) at 150 µmol L^−1^, compared to NaCl treated seedlings (Fig. 6f). Seedlings grown under stressed conditions had lower root fresh weight than unstressed plants, which was decreased by 59.67 and 63.29% under 150 and 200 mM, respectively (Fig. 6g). However, under bioSeNPs treatments, the root fresh weight was increased versus salt-stressed seedlings alone (NaCl) by 33.37, 41.77 and 62.10% (150 mM), and 42.20, 39.52 and 53.10% (200 mM), while Se (IV) significantly reduced the root fresh weight by 30.68, 51.14 and 66.71% (150 mM), and 21.64, 62.44 and 74.29% (200 mM) under 50, 100 and 150 µmol L^−1^, respectively, versus stressed plants alone (Fig. 6g).

### Impacts of Se treatments on photosynthetic pigments under two concentrations of NaCl

The results indicated significant differences (*P* < 0.05) between the treatments and photosynthetic pigments. Salinity induced negative impacts on photosynthetic pigments, which reduced total chlorophyll, chlorophyll a, chlorophyll b and carotenoids contents by 27.19, 24.48, 30.25 and 45.13% (150 mM), and 34.73, 29.83, 39.93 and 51.54% (200 mM), respectively, versus CK seedlings (Table 1). Moreover, the chlorophyll contents were affected by Se application in both forms, especially 150 µmol L^−1^ of bioSeNPs and Se (IV), which increased by 65.09 and 5.63% (chlorophyll a), 64.57 and 14.60% (chlorophyll b), 64.85 and 9.81% (total chlorophyll) under 150 mM, while 67.25 and 25.04% (chlorophyll a), 83.22 and 38.44% (chlorophyll b), 74.40 and 31.03% (total chlorophyll) under 200 mM, respectively, versus NaCl treated seedlings. On the other hand, carotenoids content was increased by 72.17 and 42.99% (150 mM), 90.52 and 54.48% (200 mM) with 150 µmol L^−1^ of bioSeNPs and Se (IV), respectively over NaCl treated seedlings (Table 1).

**Table 1:** Impacts of selenium treatments on photosynthetic pigments (mg g^−1^ FW) under two concentrations of salt stress during the early seedling stage

Traits		Chlorophyll a		Chlorophyll b		Total chlorophyll		Carotenoids	
Treatments		150 mM	200 mM	150 mM	200 mM	150 mM	200 mM	150 mM	200 mM
CK		3.628 ± 0.07^c^	3.628 ± 0.07^b^	3.428 ± 0.05^b^	3.428 ± 0.05^b^	7.056 ± 0.11^c^	7.056 ± 0.11^b^	22.29 ± 0.27^a^	22.29 ± 0.27^a^
NaCl		2.746 ± 0.08^f^	2.546 ± 0.08^d^	2.391 ± 0.05^e^	2.059 ± 0.07^e^	5.137 ± 0.08^ g^	4.605 ± 0.15^e^	12.23 ± 0.49^f^	10.80 ± 0.30^f^
50 µmol L^−1^	SeNPs	3.528 ± 0.08^c^	3.258 ± 0.09^c^	3.088 ± 0.10^c^	2.978 ± 0.07^ cd^	6.616 ± 0.02^d^	6.236 ± 0.16^ cd^	18.79 ± 0.40^ cd^	17.74 ± 0.33^cde^
	Se (IV)	3.025 ± 0.03^de^	3.285 ± 0.06^c^	2.864 ± 0.08^ cd^	3.117 ± 0.03^c^	5.888 ± 0.09^ef^	6.402 ± 0.07^c^	19.82 ± 0.20^c^	18.29 ± 0.54^ cd^
100 µmol L^−1^	SeNPs	4.075 ± 0.05^b^	3.748 ± 0.08^b^	3.726 ± 0.07^a^	3.545 ± 0.09^b^	7.801 ± 0.06^b^	7.293 ± 0.08^b^	19.84 ± 0.18^c^	18.71 ± 0.31^c^
	Se (IV)	3.153 ± 0.04^d^	3.316 ± 0.06^c^	2.971 ± 0.09^c^	2.981 ± 0.09^ cd^	6.124 ± 0.10^e^	6.297 ± 0.03^ cd^	18.57 ± 0.34^d^	17.29 ± 0.54^de^
150 µmol L^−1^	SeNPs	4.533 ± 0.08^a^	4.258 ± 0.15^a^	3.935 ± 0.05^a^	3.773 ± 0.05^a^	8.468 ± 0.11^a^	8.031 ± 0.18^a^	21.05 ± 0.28^b^	20.57 ± 0.30^b^
	Se (IV)	2.900 ± 0.09^ef^	3.183 ± 0.06^c^	2.741 ± 0.10^d^	2.851 ± 0.09^d^	5.641 ± 0.05^f^	6.034 ± 0.10^d^	17.48 ± 0.55^e^	16.68 ± 0.25^e^

Data presented are the mean ± SE of three replicates. The different letters indicate significant differences at *P* < 0.05 using Duncan's multiple range tests

### Impacts of Se treatments on osmoprotectants and MDA content under two concentrations of NaCl

**Fig. 7:**
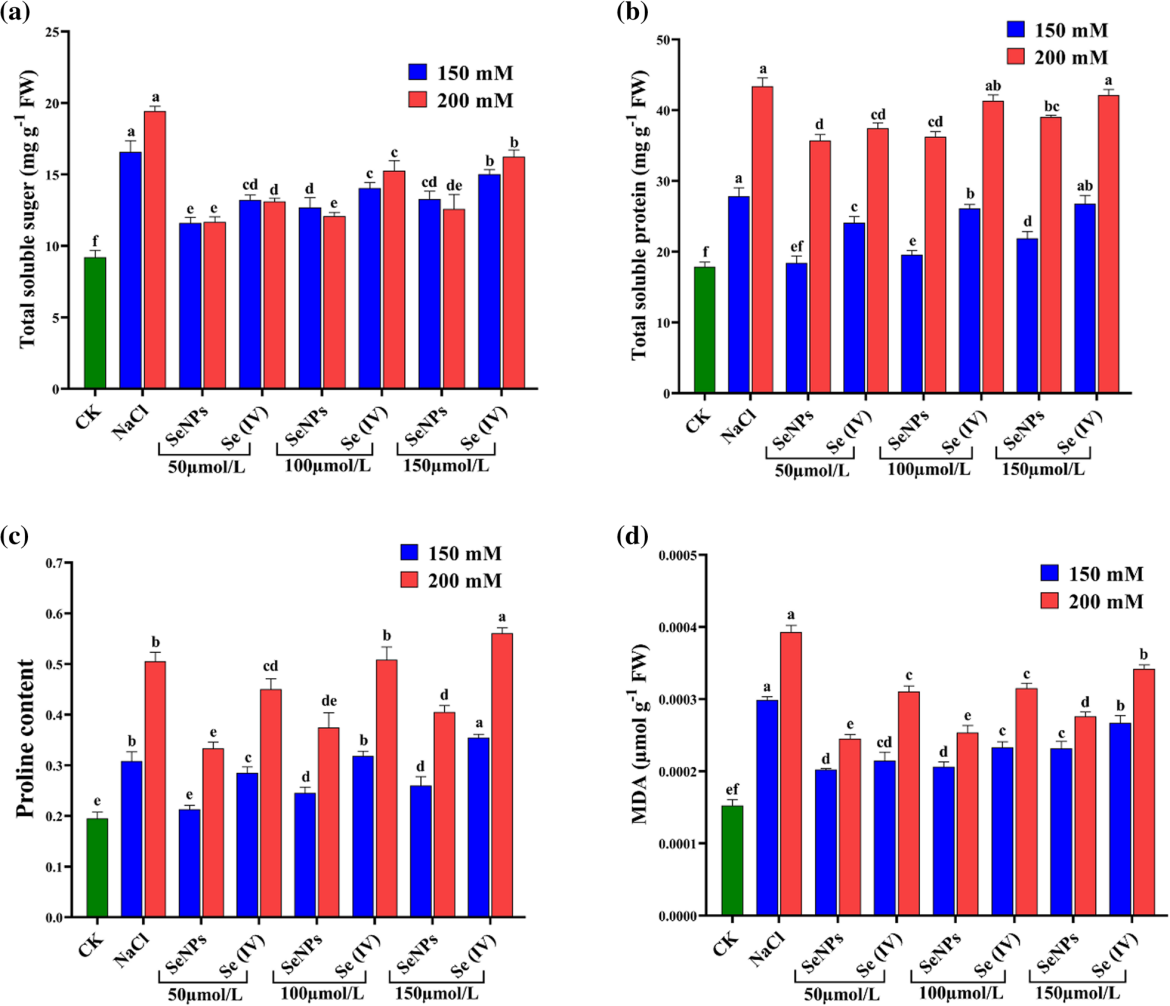
Impact of selenium treatments on **a** total soluble sugar; **b** total soluble protein; **c** proline and **d** MDA contents on Yangyou 9 under two concentrations of salt stress during the early seedling stage. Bars represent ± SE of three replicates. The different letters indicate significant differences at *P* < 0.05 using Duncan’s multiple range tests

Upon exposure to 150 and 200 mM, 80.08 and 111.2%, significantly higher total soluble sugar (TSS) content has been seen, respectively, over CK in NaCl treated seedlings. Moreover, TSS content was affected by bioSeNPs and Se (IV) application, especially 150 µmol L^−1^, which decreased TSS content by 19.86 and 9.38% (150 mM), 35.28 and 16.48% (200 mM), respectively, versus NaCl treated seedlings alone without Se treatments (Fig. 7a).

Compared with CK, total soluble protein (TSP) content was increased in NaCl treated seedlings by 55.67 and 142.6% under 150 and 200 mM, respectively. The Se treatments remarkably decreased TSP content by 33.84, 29.67 and 21.35% (150 mM), and 17.69, 16.43 and 10.01% (200 mM) with bioSeNPs, 13.48, 6.14 and 3.75% (150 mM), and 13.64, 4.73 and 2.85% (200 mM) with Se (IV) at 50, 100 and 150 µmol L^−1^, respectively, versus NaCl treated seedlings (Fig. 7b).

Proline content significantly increased by 57.86 and 158.6% under 150 and 200 mM of NaCl without Se application versus CK. Compared with NaCl treated seedlings alone without Se treatments, 150 µmol L^−1^ of bioSeNPs treatment decreased proline content by 15.78% (150 mM) and 19.78% (200 mM), while 150 µmol L^−1^ of Se (IV) treatment increased proline content by 15.02% (150 mM) and 10.94% (200 mM) (Fig. 7c).

Lipid peroxidation was determined to assess cell membrane integrity related to oxidative damage. For this purpose, the MDA level was analyzed that showed significant differences in lipid peroxidation among all treatments. Seed’s exposure of 150 and 200 mM without Se treatments extensively elevated the MDA level versus CK showed a maximum upsurge by 95.93 and 157.5%, respectively. Additionally, 150 µmol L^−1^ of Se increased MDA content by 51.70 and 75.15% (150 mM), 80.82 and 124.2% (200 mM) on bioSeNPs and Se (IV), respectively, over CK. Meanwhile, MDA content was decreased by 22.58 and 10.60% (150 mM), 29.78 and 12.91% (200 mM) with 150 µmol L^−^^1^ of bioSeNPs and Se (IV), respectively, compared to the seedling treated with NaCl alone (Fig. 7d). Besides, bioSeNPs treatments showed lower MDA content than Se (IV) treatments under salt stress.

### Impacts of Se supplementation on Na^+^, K^+^ and Na^+^/K^+^ ratio in shoots under two concentrations of NaCl

Se application decreased the Na^+^ level in the shoots and significantly elevated the uptake of K^+^ (Table 2). Under stress conditions, the Na^+^/K^+^ ratio in NaCl shoots (without Se treatments) was increased by 34.57% (150 mM) and 18.40% (200 mM) as compared to CK. Meanwhile, supplementation of Se (both forms) decreased the Na^+^ uptake and Na^+^/K^+^ ratio as well as increased the K^+^ uptake, especially, 150 µmol L^−1^ of bioSeNPs and Se (IV), which decreased the Na^+^ content by 35.29 and 26.88% (150 mM), 34.64 and 24.34% (200 mM), and increased the K^+^ content by 203.3 and 136.7% (150 mM), 414.2 and 233.6% (200 mM), as well as reduced the Na^+^/K^+^ ratio by 78.66 and 69.10% (150 mM), 87.92 and 77.32% (200 mM), respectively, versus NaCl. Ultimately, bioSeNPs showed significant positive effects strikingly stronger on the mineral uptake (Table 2).

**Table 2:** Impact of selenium supplementation on Na^+^, K^+^ and Na^+^/K^+^ ratio in shoots under two concentrations of salt stress during the early seedling stage

Traits		Na^+^ mg/g		K^+^ mg/g		Na^+^/K^+^	
Treatments		150 mM	200 mM	150 mM	200 mM	150 mM	200 mM
CK		3.80 ± 0.33^e^	3.80 ± 0.33^f^	7.77 ± 0.17^f^	7.77 ± 0.17^f^	0.49 ± 0.04^b^	0.49 ± 0.04^b^
NaCl		36.47 ± 0.65^a^	46.76 ± 0.71^a^	2.37 ± 0.29^e^	0.98 ± 0.59^e^	15.39 ± 0.01^a^	47.71 ± 0.26^a^
50 µmol L^−1^	SeNPs	26.25 ± 0.52^c^	35.23 ± 0.28^c^	5.02 ± 0.59^c^	2.83 ± 0.35^b^	5.23 ± 0. 21^def^	12.45 ± 0.12^de^
	Se (IV)	30.57 ± 0.32^b^	36.54 ± 0.26^c^	3.33 ± 0.64^d^	2.01 ± 0.68^d^	9.18 ± 0.22^c^	18.18 ± 0.15^c^
100 µmol L^−1^	SeNPs	25.22 ± 0.57^ cd^	32.47 ± 0.91^d^	5.56 ± 0.58^b^	3.68 ± 0.08^b^	4.54 ± 0.31^ef^	8.820 ± 0.19^ef^
	Se (IV)	29.34 ± 0.71^b^	38.23 ± 0.35^b^	4.04 ± 0.54^d^	2.52 ± 0.68^c^	7.26 ± 0.44^ cd^	15.17 ± 0.35^ cd^
150 µmol L^−1^	SeNPs	23.60 ± 0.61^d^	30.56 ± 0.55^e^	7. 19 ± 0.49^a^	5.04 ± 0.70^a^	3.28 ± 0.32^f^	6.060 ± 0.19^f^
	Se (IV)	26.67 ± 0.93^c^	35.38 ± 0.39^c^	5.61 ± 0.62^c^	3.27 ± 0.33^b^	4.75 ± 0.21^de^	10.82 ± 0.27^de^

Data presented are the mean ± SE of three replicates. The different letters indicate significant differences at *P* < 0.05 using Duncan's multiple range tests

### BioSeNPs and Se (VI) affected Se pathway-related genes in *B. napus* root tissues under 150 µmol L^−1^

**Fig. 8:**
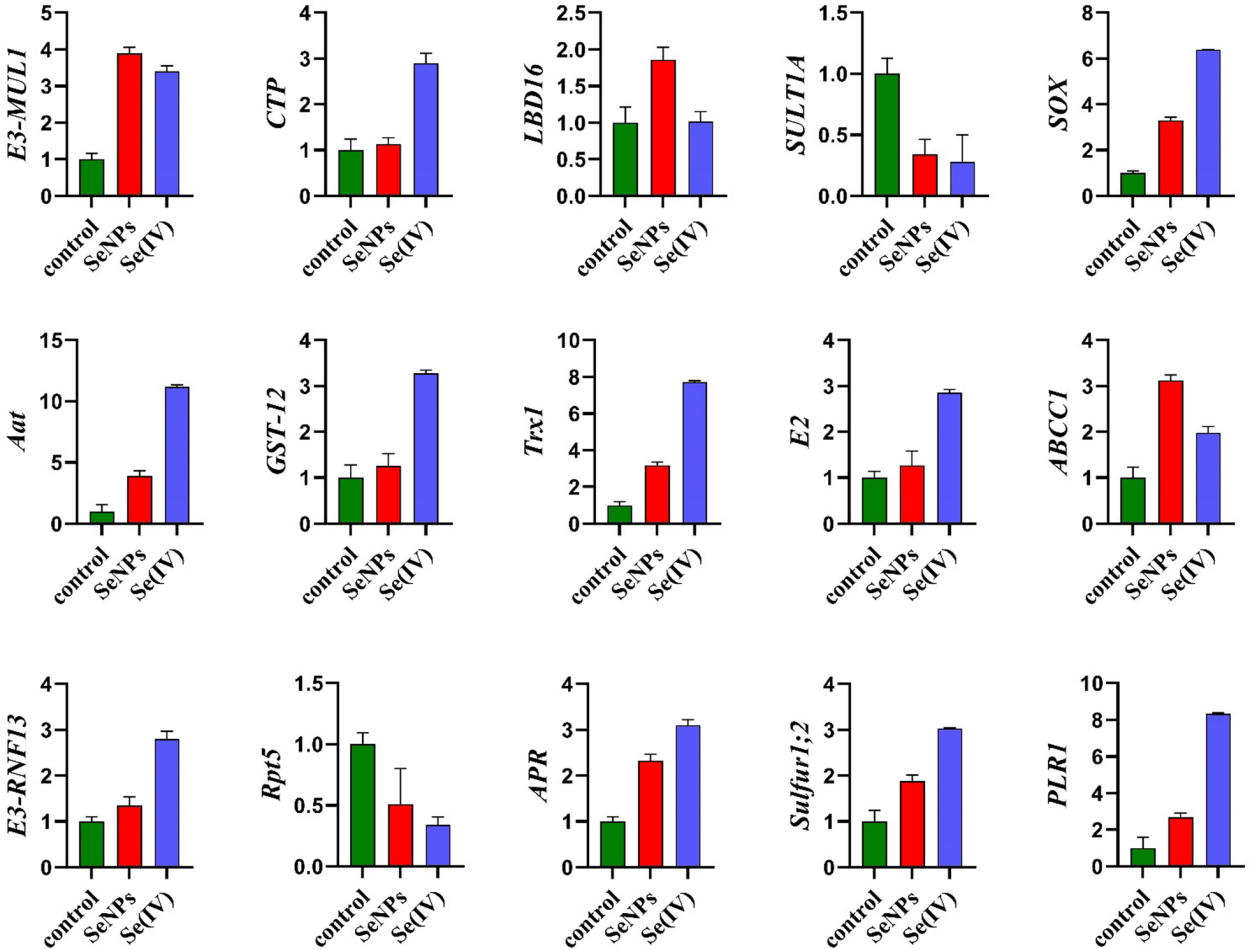
Higher doses of SeNPs and Se (IV) differentially changed selenium pathway-related genes in rapeseed root tissue. Seven-day-old root tissues treated with 150 µmol L^−1^ of SeNPs and Se (IV) were used to quantify the expression levels. β-ACTIN was used as a reference gene. Bars represent ± SD of three replicates

The transcription levels of Se pathway-related genes were determined to evaluate the impact of bioSeNPs and Se (IV) on root tissues of *B. napus.* In seedlings-root, the expression of sulfite oxidase gene (*SOX*) was up-regulated under bioSeNPs and Se (IV) treatments by 3.30 and 6.39-fold, respectively (Fig. 8). Our results verified that Se (IV) could transform into Se (VI) form, which persists in the metabolic pathway. Also, the storage function of the vacuole is of great importance, which was verified through genes upregulation in the metabolism.

Also, Se (IV) stress enhanced the gene expression of both *Aat* and *PLR1* by 11.19 and 8.33-fold, respectively, which are significant genes for the metabolism of amino acids, and might participate in the determination of the pyridoxal phosphate content in the cell. Meanwhile, bioSeNPs treatment induced the expression of *LBD16* by a 1.86-fold relative to control (Fig. 8). Our findings illustrated that bioSeNPs could improve lateral root production in plants, one of the main architectural determinants for root development. Previously NPs were well documented to have essential functions in growth by increasing the expression of *LBD16*, which acts downstream of the auxin influx carriers AUX1 and LAX1 in the regulation of lateral root initiation and development; thus, it regulates the developmental processes in plants [49].

Mitochondrial located gene *E3* ubiquitin-protein ligase *MUL1* was highly expressed along with *E3-RNF13* and ubiquitin-conjugating enzyme *E2* genes, which are important for the ubiquitin–proteasome pathway (UPP). Furthermore, sulfate transporter 1;2 (Sultr1;2) is an essential protein for sulfate and selenate transportation, which is increased by 1.88 and 3.03-fold under bioSeNPs and Se (IV) treatments, respectively, which in turn played an important role in selenium uptake repression. The expression level of *APR1* recorded 2.33 and 3.10-fold, while the expression level of *SULT1A* decreased by 0.34 and 0.28-fold under bioSeNPs and Se (IV), respectively (Fig. 8).

BioSeNPs and Se (IV) up-regulated the expression level of *Trx 1* by 3.18 and 7.72-fold, respectively (Fig. 8), which played significant roles in detoxifying ROS and the maintenance of cellular redox homeostasis via structural alterations of target proteins [50]. Consequently, the Se (IV) treatments negatively affected the root growth of rapeseed seedlings versus bioSeNPs and relative control.

### Effect of bioSeNPs and Se (VI) on Se pathway-related genes in *B. napus* shoot tissues under 150 µmol L^−1^

**Fig. 9:**
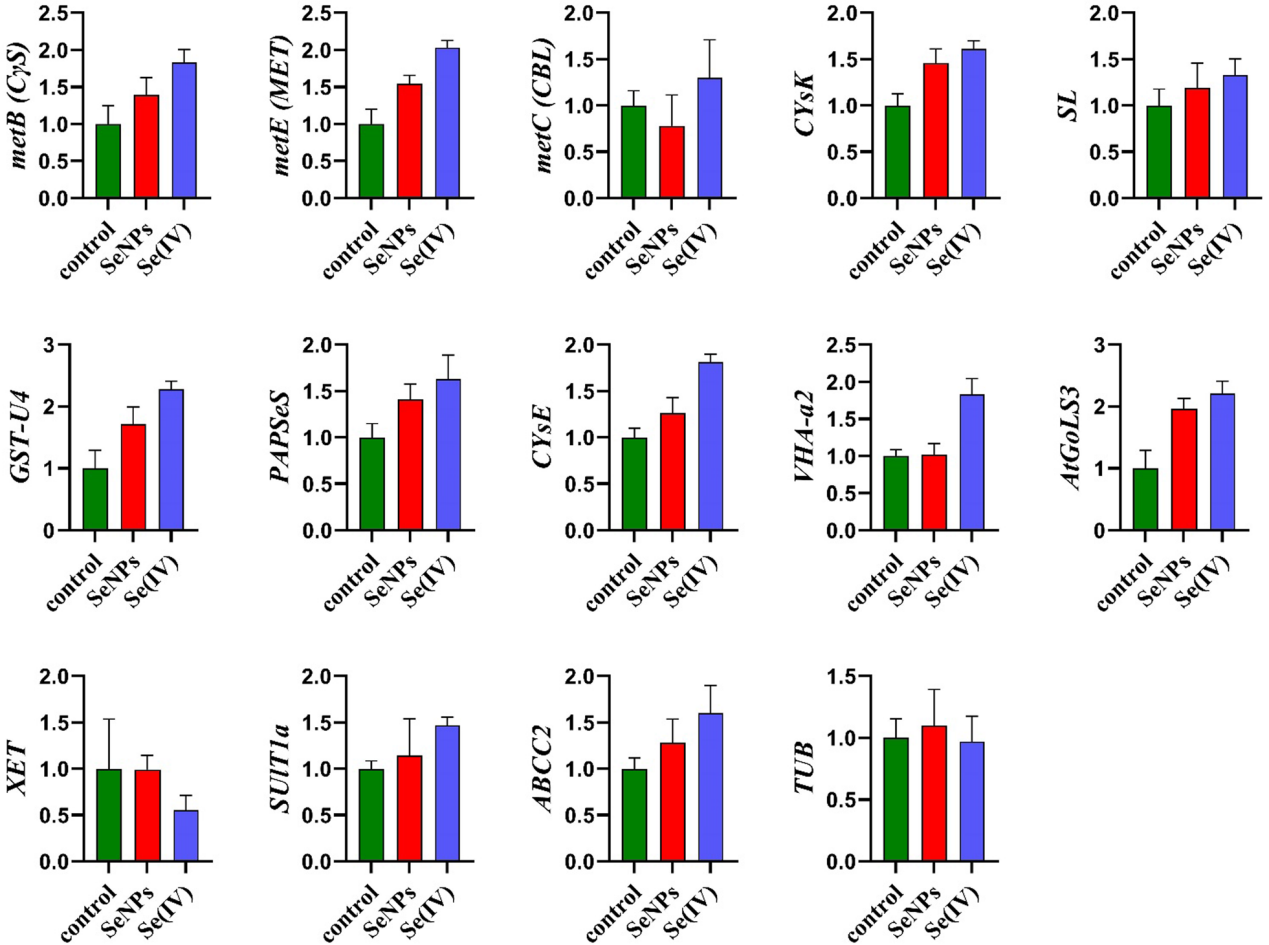
Higher doses of SeNPs and Se (IV) differentially changed selenium pathway-related genes in rapeseed shoot tissues. Seven-day-old shoot tissues treated with 150 µmol L^−1^ of SeNPs and Se (IV) were used to quantify the expression levels. β-ACTIN was used as a reference gene. Bars represent ± SD of three replicates

To explore the impact of bioSeNPs and Se (IV) on Se pathway-related genes in *B. napus* shoot tissue, we have selected *ABCC2* from the C subfamily of the ATP-binding cassette transporters (ABCC) and *GST-u4* from the glutathione S-transferase family, which was increased by 1.28- and 1.71-fold under bioSeNPs treatmentand 1.60 and 2.28-fold under Se (IV) treatment, respectively. Furthermore, Se (IV) treatment increased the expression levels of *CγS*, *MET,* and *CBL* by 1.83, 2.03 and 1.30-fold, respectively. These three enzymes are critical in the methionine biosynthetic pathway (Fig. 9). Meanwhile, *CysK*, *CysE* and *SL* increased under Se treatments by 1.46, 1.26 and 1.19-fold (bioSeNPs), 1.61, 1.81 and 1.33-fold (Se (IV)), respectively.

*SULT1A* is a member of the sulfotransferase (SOT) protein family, which significantly participates in the selenation pathway [51]. The transcription level of *SULT1A* was increased by 1.14-fold (bioSeNPs) and 1.47-fold (Se (IV) in the shoot tissues (Fig. 9). *AtGoLS3* belongs to the galactinol synthase (*GolS*) family is a key regulator in raffinose family oligosaccharides (RFOs) synthesis that promotes plant stress tolerance under various abiotic stresses [52]. Regarding our results, the *AtGoLS3* gene expression under selenium treatments was increased by 1.96 and 2.21-fold under bioSeNPs and Se (IV), respectively (Fig. 9). These results suggested that *AtGoLS3* was enhanced under abiotic and metal stress.

However, the expression level of *XET* was reduced by 0.55-fold under Se (IV), while slightly reduced under bioSeNPs treatments (0.55-fold), it contributed to the wall stress relaxation, strengthening, gravitropism and increased the creep of cellulose xyloglucan composites in the cell wall during biosynthesis [53]. The existing gene on mature phagosomes *VHA-a2* was enhanced by 1.02- and 1.83-fold in the shoot tissues under bioSeNPs and Se (IV) treatments, respectively. At the same time, the changes in the expression level of *TUB* gene increased by 1.10- and 0.97-fold at bioSeNPs and Se (IV), respectively (Fig. 9).

## Discussion

Nanoparticles including metallic elements such as CuNPs, FeNPs, CeNPs, TiNPs, AgNPs and ZnNPs interact with plant cells at the physicochemical level based on their surface properties, giving each element a typical response by enhancing the uptake of beneficial or essential elements to the plant [54–56]. Nowadays, SeNPs has attracted many researchers, owing to their unique physiochemical properties and availability, which increased the application of Se in the agricultural field, particularly under biotic and abiotic stresses. Our goal is to investigate the effect of two forms of Se (bioSeNPs and Se (IV)) on germination and growth under normal and salt stress conditions using higher Se doses in *B. napus*.

### BioSeNPs positively affect seed germination and seedling growth of *B. napus* during the early seedling stage

In our study, bioSeNPs possess lower phytotoxicity than Se (IV) in seedlings under normal and salt stress conditions. It may significantly affect plant development and seed germination, which impact seedling growth under higher Se doses. This result indicated that the application of bioSeNPs induced marked proliferation in growth parameters. Furthermore, it was reported that plants can easily uptake and transport NPs [57], suggesting that bioSeNPs might be interacting with plants at the cellular and subcellular levels after entering the plant cell and promoting changes in morpho-physiochemical attributes [58, 59].

Moreover, SeNPs positively impacted several physiochemical mechanisms and improved shoot and root development in tomato plants [48], encouraging organ production and root development in tobacco [60]. These studies, as mentioned earlier, could explain our findings of bioSeNPs enhancing the seedling growth and biomass, which was observed in comparison with Se (IV).

Contrarily, Se (IV), especially 150 µmol L^−1^, declined the growth traits under both conditions (normal and salt stress conditions), which caused chlorosis and stunted plant growth, leaves withering, dryness and premature death [61, 62]. Moreover, higher doses of Se (IV) induced adverse effects on seedling development, which increased ROS accumulation, deterioration in total chlorophyll contents, and un-specified substitution of sulfate ion (SO_4_^2−^) in S-containing substances and protein that inhibited the growth parameters [63, 64].

### Nano-Se positively affect the photosynthetic pigments and osmolality components

Photosynthetic pigments are essential energy sources of plant biological systems, which are a vital index of photosynthesis and any alteration causes a parallel effect on metabolism [57]. Interestingly, in our study, bioSeNPs significantly increased the chlorophyll contents compared to Se (IV) and untreated seedlings (Control) under normal and stress conditions. We noticed that 150 µmol L^−1^ showed the highest value of chlorophyll contents due to higher protection of photosynthetic pigments with higher Se concentration. However, bioSeNPs 50 and 100 µmol L^−1^ showed a non-significant difference compared to each other. Furthermore, previous studies reported that the effect of NPs on the chlorophyll contents is concentration-dependent [54, 65, 66]. Therefore, different concentrations of NPs are of great importance for the various effects on plant's physiological processes [67]. A higher chlorophyll contents might be related to bioSeNPs induced protection of certain chloroplast enzymes by improving antioxidant enzymatic capacity [68]. Se plays a role as the catalytic center of selenoproteins like GPx and scavenges the free radicals that protect the photosynthetic pigment [2]. On the other side, higher doses of Se (IV) declined the chlorophyll contents, which may induce some oxidative strain and lead to peroxidation of chloroplast membrane and photosynthetic degeneration [69]. Additionally, the higher concentration of Se resulted in a lower photosynthetic capacity, presumably attributable to the chlorophyll degeneration induced by the decrease in carotenoids [70, 71].

Osmolytes are essential to improve the defense system under different stresses, supplying metabolites and energy via several physiochemical operations [72]. BioSeNPs improve osmotic substances through osmotic potential maintenance within the cytoplasm and vacuoles during the metabolic processes and provide cellular protection against ROS accumulation. In general, the metallic NPs may improve the light absorption through chloroplast by increasing the gene expression response to light-harvesting complex II, raising the osmolyte contents [73, 74].

Additionally, the plant growth performance is mainly correlated with the water status. The plant responds to water shortage by accumulating several osmotic protectants such as proline [75, 76]. In this investigation, we noticed a dramatic elevation in proline contents under Se (IV) than bioSeNPs, which may explain the exceptional ability of rapeseed seedlings to resist selenium-induced water loss; moreover, higher proline content indicated stress level [77]. Generally, bioSeNPs non-significantly affected the proline content under both conditions, which explained the critical role of proline in plants to avoid the harmful effects of ROS [78].

### BioSeNPs induced plant defense system under normal and stress conditions

Malondialdehyde (MDA) is produced by the oxidative degradation of cellular membrane, an indicator of membrane damage, considered another parameter reflecting the cell’s vitality [20]. In the current study, the MDA content was increased in treated seedlings with Se (IV), which may cause specific stress and thus induce enzymatic and non-enzymatic antioxidants [77, 79]. In contrast, bioSeNPs recorded a slight increment in MDA content over the relative control because of the specific response of rapeseed seedlings to nano-Se by improving photosynthesis besides antioxidant enzyme activity.

ROS leads to oxidative stress and disrupts various cellular structures in plants [80]. The equilibrium between production and scavenging of ROS decides either oxidative damage or stress signalling [1], and the excessive ROS production is responsible for substituting sulfur, preventing methylation and Se-toxicity [2]. Furthermore, SOD and CAT activities were not synchronized due to the continuous elevation of SOD and the reduction of CAT activities, which accelerated the generation of H_2_O_2_ under Se treatments in rapeseed seedlings, especially under Se (IV) compared to bioSeNPs. Besides, bioSeNPs affected antioxidant enzymes efficiencies, thus protecting the plants and enhancing the growth, which indicated the free radicle detoxification [81–83], antioxidant defense system stimulation and ROS quenching by NPs [57]. Meanwhile, Se (IV) toxicity was potentiated by impairment of oxidative metabolism and higher productions of ROS, as evidenced by higher accumulation of H_2_O_2_, O_2_^•−^ and lipid peroxidation that caused plasma membrane injury [70].

Our investigation showed that bioSeNPs application-maintained homeostasis in the cell by inducing the gene expression of antioxidant enzymes related genes owing to higher POX activity. The following activities of SOD and CAT lessened the ROS damage by O_2_^•−^ → H_2_O_2_ → H_2_O progression due to the protective role of Se under oxidative stress [84, 85], suggesting that nano-treatment can alleviate oxidative damages of metal toxicity and salt stress by decreasing the excessive lipid peroxidation, which improves cellular integrity. Contrarily, a higher dose of Se (IV) influenced the transcription level and activity of antioxidant enzymes by promoting SOD and reducing CAT activities, ultimately excessive ROS accumulation [70].

### Supplementation of bioSeNPs reduced the Na^+^ toxicity during the early seedling stage

A plant’s ability to maintain both K^+^ and Na^+^ homeostasis is an essential trait indicating its salinity stress tolerance. Massive over-accumulation of Na^+^ in the cytosol leads to Na^+^ toxicity and causes K^+^ efflux from the cytosol to apoplast. BioSeNPs application increased K^+^ level with reduction of Na^+^ level under two concentrations of NaCl (150 and 200 mM) during the early seedling stage, which promotes the regulation of growth by inference to antioxidant metabolism and cellular stress signalling [86]. It could be concluded that the enhancement in rapeseed growth, total chlorophyll and mineral contents of seedlings exposed to salt conditions with bioSeNPs application may be due to reducing Na^+^ content in leaves, consequently reducing Na^+^/K^+^ ratio, osmotic adjustment, ionic balance and antioxidant enzyme activities [87]. NPs elevate the expression of Na^+^/H^+^ antiport and tonoplast H^+^-ATPase at the root membranes and shorten the root apoplast barrier, thus limiting Na^+^ translocation to shoot tissues and reducing Na^+^ toxic effects [88, 89], hence improving salt tolerance. Se application alleviates salinity stress by enhancing PSII function and decreasing Na^+^ content in the shoot via binding of Na^+^ to the root cell wall, ultimately reducing the accumulation of Na^+^ ions in plant organs [90].

K^+^ is a co-factor required to activate more than 50 enzymes [91] and plays a vital role in cytosolic pH homeostasis, protein synthesis and cell activities, including stomatal opening and closure [92]. Conclusively, we found that bioSeNPs treated seedlings showed a better ability to maintain total leaf K^+^ content than Se (IV) under salt stress. These results confirmed that bioSeNPs helped rapeseed seedlings to maintain a better leaf Na^+^/K^+^ ratio, thus better performance under salinity stress. Overall, this work suggests that retaining the leaf Na^+^/K^+^ ratio is a component of the mechanisms behind SeNPs enabled better plant salt tolerance.

### Se (VI) as a fundamental compound in the Se pathway

**Fig. 10:**
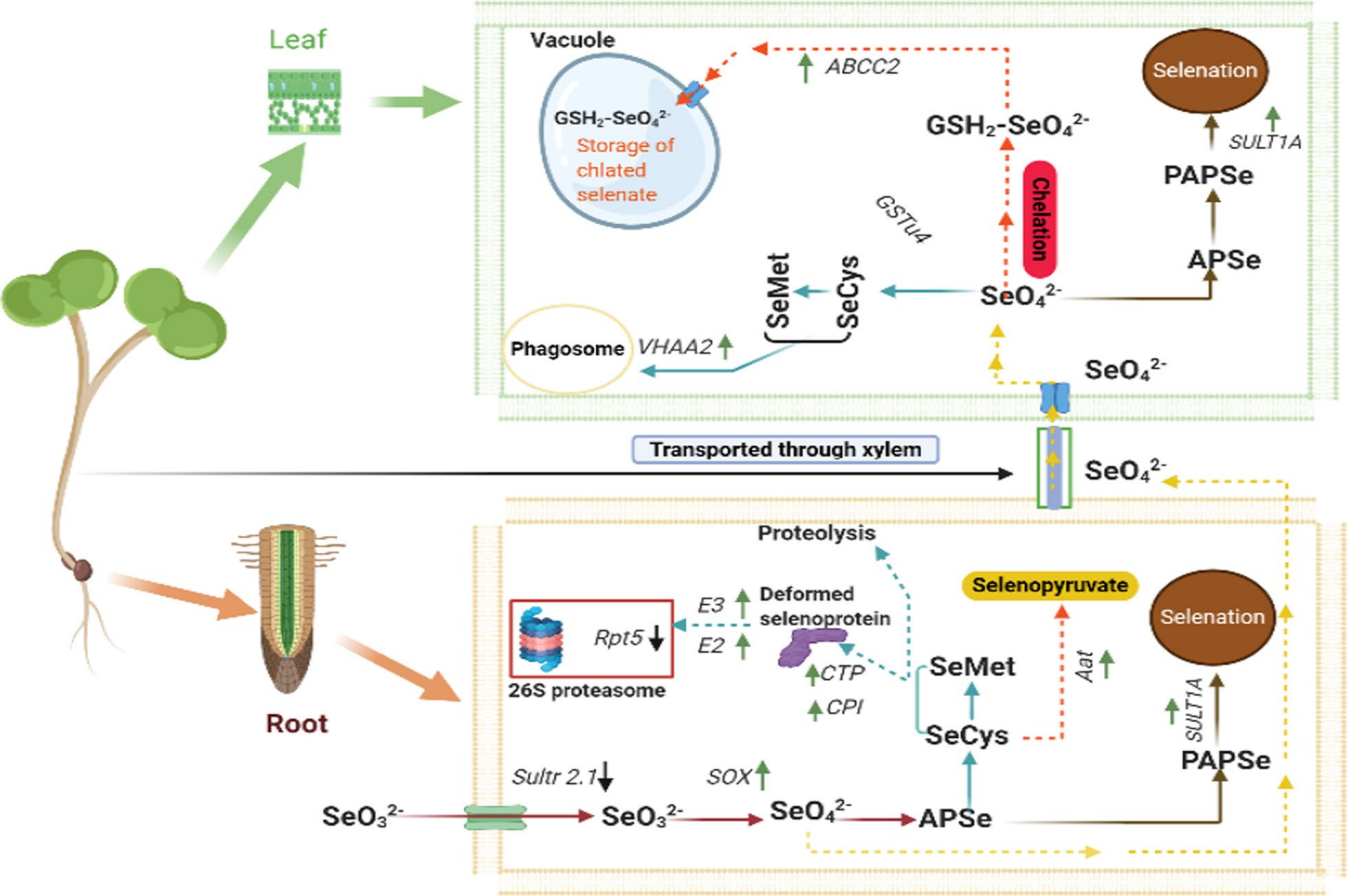
Conclusion of Se detoxification mechanisms in rapeseed seedlings

Most of the plants nonspecifically uptake selenate using sulfate transporters as Se, which is similar to S in terms of its chemical characteristics [93, 94]. To convert Se (VI) into Se (IV), the performance of both APS and APR enzymes is essential. APS enzyme intercedes the Se (VI) linkage with ATP, resulting in adenosine phosphoselenate (APSe), which is converted into Se (IV) via APR [6]. Furthermore, the rise in SOX level confirmed that Se (IV) might be transformed into Se (VI), then integrated to ATP through APS, converted to Se (IV) through APR, then into selenide; finally, integrated into SeCys in rapeseed seedlings specifically in root tissues (Fig. 10).

### Vacuole plays a vital role under high doses of Se

The formation of APSe, followed by the increase of GST-u4, facilitated the transformation of Se (VI) to GSH and glutathione-S conjugate (GS-X). The subfamily C-CFTR/MRP of the ATP-binding cassette superfamily was first identified as phytochelatin transporters in the sequestration of toxic elements in the vacuole [95, 96]. Therefore, we deduced that glutathione-derived peptides partially chelated Se (VI) with glutathione sulfurtransferase (GST), then translocated to the vacuole through MRP2 to protect the cell from the deleterious Se effects and Se sequestration in different tissues (shoot and root). Our findings proposed that vacuole storage functions are vital to cope with high concentrations of Se in *B. napus* seedlings (Fig. 10).

### Transamination plays a significant role to protect plant cells against Se toxicity

SeCys is one of the critical factors for Se toxicity, and the misincorporation of SeCys affects the Se-detoxification ability of plants [97]. SeCys methyltransferase is the main factor in SeCys methylation, using *CyS* to rehabilitate into SeMet, and it is specifically degraded or oxidized through *CpNifS* and *SL*, respectively [6]. Our results confirmed that the up-regulation of *CγS*, *CBL* and *MET* is responsible for SeCys-SeMet conversion in the shoot tissues. While, the higher expression levels of *PLR1* and *Aat* in the root tissues are also located in the cysteine and methionine metabolic pathway [98], which concluded SeCys deamination by *Aat* as a remarkable way for Se tolerance (Fig. 10).

### Selenation is a substantial way for Se detoxification

Selenation is one of the most important pathways for Se detoxification, similar to the sulfation process that can reduce SeCys biosynthesis. On the other side, *APR* is considered a critical enzyme for reducing selenate and sulfate [2]. Furthermore, our observation suggested that the Se (VI) either chelated by *GSH* then entered in the vacuole or shifted to a phenolic hydroxy group by *APSe*, *PAPSeS* and *SULT1A*, ultimately formed selenocompound substrates [51]. Our conclusion reported that the selenation process also happened at high selenium doses in the root tissues. The selenation activity was greater in shoots than roots due to the higher expression level of *SULT1a* under Se toxicity. Correspondingly, our results supported that selenation is a more functional method of Se detoxification in rapeseed seedlings (Fig. 10).

### Selenoproteins degradation is a noteworthy pathway for Se detoxification

The SeMet and SeCys are selenoamino acids that might be misincorporated with proteins. Cysteine occupies an essential position in structural integrity and functional maintenance of protein. Moreover, it contributes to various processes such as enzymatic reactions, redox homeostasis, folding of the protein and metal detoxification. Protein deformation probably occurs by substituting SeCys in non-specific selenoproteins, leading to diselenide linkage or a mixed selenide-sulfide linkage, which are different in characteristics. Therefore, the non-specific SeMet accumulation is reported as less detrimental than highly reactive SeCys [64]. Chaperone-mediated processes induced the formation of deformed or malformed selenoproteins, and the proteolysis of irreparable proteins through the lysosome or the ubiquitin–proteasome pathway (UPP) can also occur. The processes of nullifying the production of selenoproteins are associated with Se tolerance in plants, which supports our findings [97, 99]. The changes in the expression levels of *E3* ubiquitin-protein ligase *MUL1*, *E2* ubiquitin-conjugating enzyme *RNF13* and cysteine-type peptidase in rapeseed roots, and *VHA-a2* in rapeseed shoots, in addition to the changes in *Rpt5* as an essential subunit for assembly of the 26S proteasome, indicate that the selenoproteins disintegration is a crucial way for Se tolerance, which is rhythmic with [97, 99, 100] (Fig. 10).

## Conclusion

We aimed to investigate the effect of bioSeNPs and Se (IV) on morpho-physiochemical attributes under normal and salt stress conditions, besides the molecular mechanisms and genes involved in Se detoxification during the early seedling stage. Our experiments provide evidence that the morpho-physiochemical response of rapeseed to bioSeNPs was generally more significant than the relative control and Se (IV) treatments under normal and salt stress conditions. Furthermore, our investigation highlights the considerable efficacy of bioSeNPs to improve seed germination and seedling growth, elevate photosynthesis capacity, secondary metabolism and defense system ability. Our work suggests that retaining the leaf Na^+^/K^+^ ratio is a component of the mechanisms behind bioSeNPs enabled better plant salt tolerance. Additionally, our results supported that selenation is a more functional method of Se detoxification and vacuole storage is vital for coping with higher Se doses in rapeseed seedlings. The oxidation and transamination of SeCys in rapeseed roots and the conversion of SeCys into SeMet in rapeseed shoots are essential processes for Se detoxification, and selenoproteins degradation is an important way to increase Se tolerance in rapeseed seedling. Taking knowledge gaps into account, these comprehensive comparative data can be helpful to gain novel insights into the benefits or the risk associated with bioSeNPs or Se (IV) function in agriculture and the different pathways of Se detoxification. Additionally, our findings suggested that bioSeNPs is a valuable alternative method for the remediation of reduced growth under abiotic stresses with a novel and eco-friendly technique besides low-cost investment as an advantage, which is a promising material in ameliorating the rapeseed growth and development.

## Supplementary Information

**Additional file 1: Fig. S1.** Selenium pathway in plants. **Fig. S2.** Preparation and purification of nano selenium (SeNPs).** Fig. S3.** Effect of different concentrations of SeNPs and Na_2_SeO_3_ (0, 50, 100 and 150 µmol/L) on (a) shoot dry weight (g) and (b) root dry weight (g) on rapeseed seedlings. Bars represent ± SE of three replicates. The difference in letters indicate significant differences at *P* < 0.05 using Duncan's multiple range tests.** Fig. S4**. (a): Heat map, and A Pearson’s correlation of (b): SeNPs and (c) Se (IV) showing the effects of different doses of SeNPs and Se (IV) (0, 50, 100 and 150 µmol/L) on the morpho-physiochemical attributes in rapeseed seedlings. Color scale corresponds to the logarithmic transformation of measured values (higher levels are shown in blue, lower levels in red and intermediate levels in dark colors for both blue/red). FG%: final germination percentage; GR: germination rate; VI (I): vigor index I; VI (II): vigor index II; ShL: shoot length; RL: root length; ShFW: shoot fresh weight; RFW: root fresh weight; ShDW: shoot dry weight; RDW: root dry weight; Chl a: chlorophyll a; Chl b: chlorophyll b; TC: total chlorophyll; C: carotenoids content; TSS: total soluble sugar; TP: total protein; MDA: Lipid peroxidation; P: proline content; SOD: super oxidase dismutase; POD: peroxidase; CAT: catalase; APX: ascorbate peroxidase and GR: glutathione reductase. **Fig**. **S5**. Impacts of selenium treatments on (a) shoot dry weight (g), and (b) root dry weight (g) on Yangyou 9 under two concentrations of salt stress during the early seedling stage. Bars represent ± SE of three replicates. The different letters indicate significant differences at *P* < 0.05 using Duncan's multiple range tests.** Table S1.** Sequences of primers used in this study.

## Data Availability

Data is contained within the article.
