# Supplementary material for: Comparative efficacy of bio-selenium nanoparticles and sodium selenite on morpho-physiochemical attributes under normal and salt stress conditions, besides selenium detoxification pathways in Brassica napus L
Source: J Nanobiotechnology. 2022 Mar 27;20:163. doi: 10.1186/s12951-022-01370-4 (PMC8962572; doi:10.1186/s12951-022-01370-4)
Supplement: Supplementary file 1 — Additional file 1: Fig. S1. Selenium pathway in plants. Fig. S2. Preparation and purification of nano selenium (SeNPs). Fig. S3. Effect of different concentrations of SeNPs and Na2SeO3 (0, 50, 100 and 150 µmol/L) on (a) shoot dry weight (g) and (b) root dry weight (g) on rapeseed seedlings. Bars represent ± SE of three replicates. The difference in letters indicate significant differences at P < 0.05 using Duncan's multiple range tests. Fig. S4. (a): Heat map, and A Pearson’s correlation of (b): SeNPs and (c) Se (IV) showing the effects of different doses of SeNPs and Se (IV) (0, 50, 100 and 150 µmol/L) on the morpho-physiochemical attributes in rapeseed seedlings. Color scale corresponds to the logarithmic transformation of measured values (higher levels are shown in blue, lower levels in red and intermediate levels in dark colors for both blue/red). FG%: final germination percentage; GR: germination rate; VI (I): vigor index I; VI (II): vigor index II; ShL: shoot length; RL: root length; ShFW: shoot fresh weight; RFW: root fresh weight; ShDW: shoot dry weight; RDW: root dry weight; Chl a: chlorophyll a; Chl b: chlorophyll b; TC: total chlorophyll; C: carotenoids content; TSS: total soluble sugar; TP: total protein; MDA: Lipid peroxidation; P: proline content; SOD: super oxidase dismutase; POD: peroxidase; CAT: catalase; APX: ascorbate peroxidase and GR: glutathione reductase. Fig. S5. Impacts of selenium treatments on (a) shoot dry weight (g), and (b) root dry weight (g) on Yangyou 9 under two concentrations of salt stress during the early seedling stage. Bars represent ± SE of three replicates. The different letters indicate significant differences at P < 0.05 using Duncan's multiple range tests. Table S1. Sequences of primers used in this study. [file 12951_2022_1370_MOESM1_ESM.docx]

| 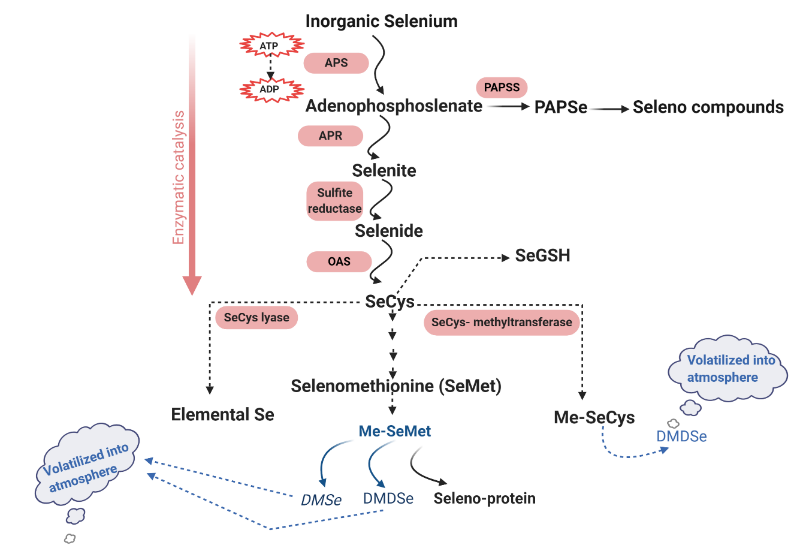 |
| --- |

**Fig. S1.** Selenium pathway in plants.

| 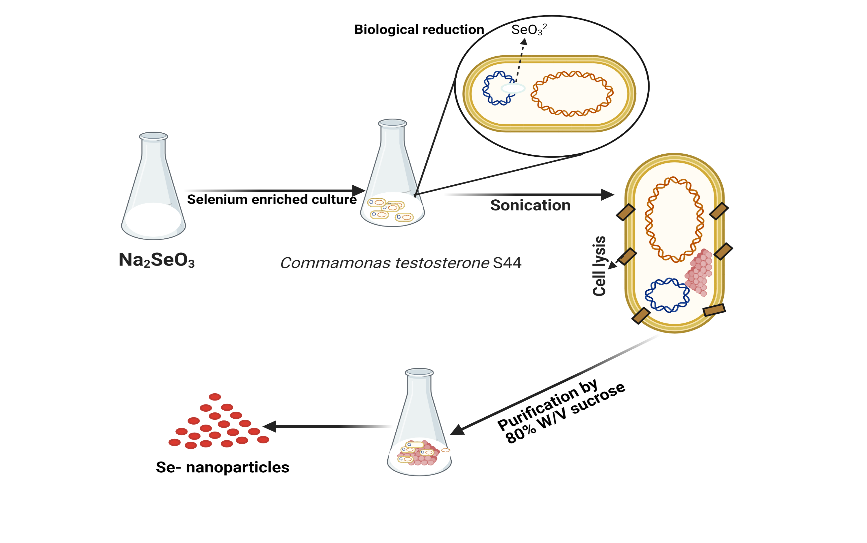 |
| --- |

**Fig. S2.** Preparation and purification of nano selenium (SeNPs).

| 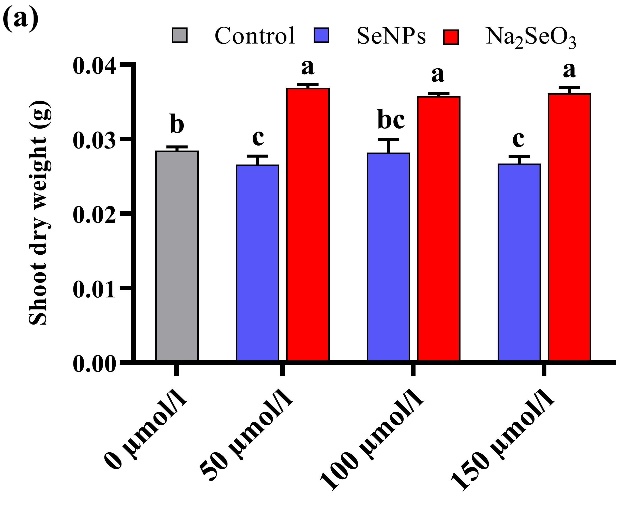 | 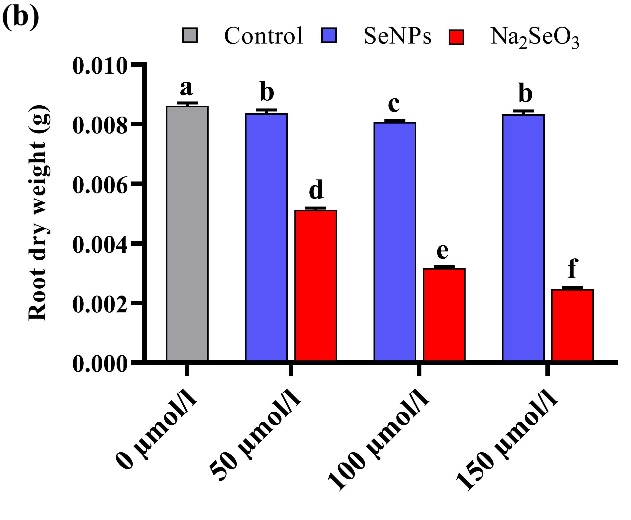 |
| --- | --- |

**Fig. S3.** Effect of different concentrations of SeNPs and Na_2_SeO_3_ (0, 50, 100 and 150 µmol/L) on (a) shoot dry weight (g) and (b) root dry weight (g) on rapeseed seedlings. Bars represent ± SE of three replicates. The difference in letters indicates significant differences at (*P* < 0.05) using Duncan's multiple range tests.

| 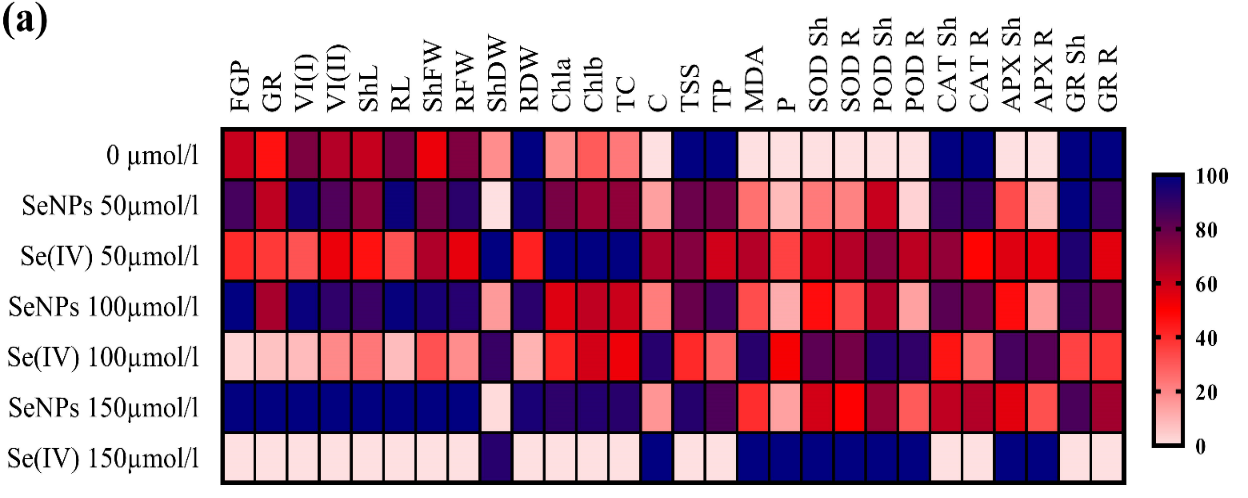 | |
| --- | --- |
| 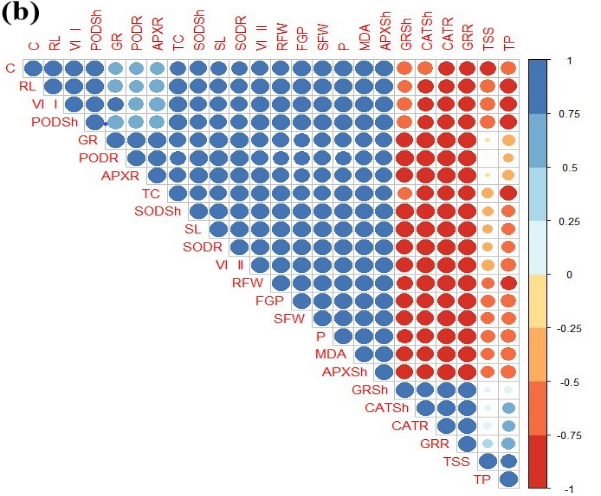 | 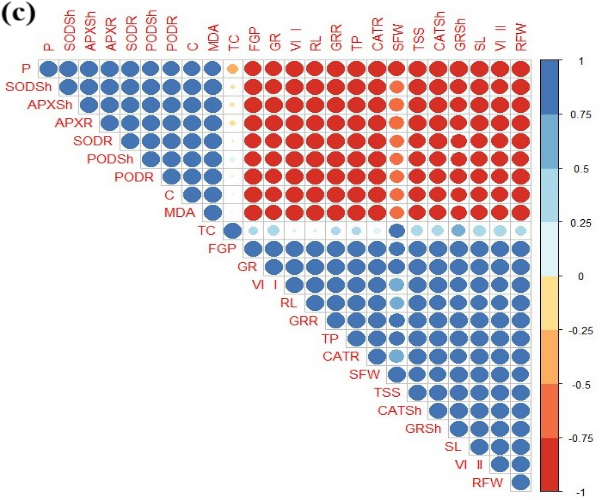 |

**Fig. S4**. (a): Heat map, and A Pearson’s correlation of (b): SeNPs and (c) Se (IV) showing the effects of different doses of SeNPs and Se (IV) (0, 50, 100, and 150 µmol/L) on the morphophysiological parameters in the seedling of rapeseed. Color scale corresponds to the logarithmic transformation of measured values (higher levels are shown in blue, lower levels in red and intermediate levels in dark colors for both blue/red). FG%: final germination percentage; GR: germination rate; VI (I): vigor index I; VI (II): vigor index II; ShL: shoot length; RL: root length; ShFW: shoot fresh weight; RFW: root fresh weight; ShDW: shoot dry weight; RDW: root dry weight; Chl a: chlorophyll a; Chl b: chlorophyll b; TC: total chlorophyll; C: carotenoid contents; TSS: total soluble sugar; TP: total protein; MDA: Lipid peroxidation; P: proline content; SOD: super oxidase dismutase activity; POD: peroxidase activity; CAT: catalase activity; APX: ascorbate peroxidase and GR: glutathione reductase.

| 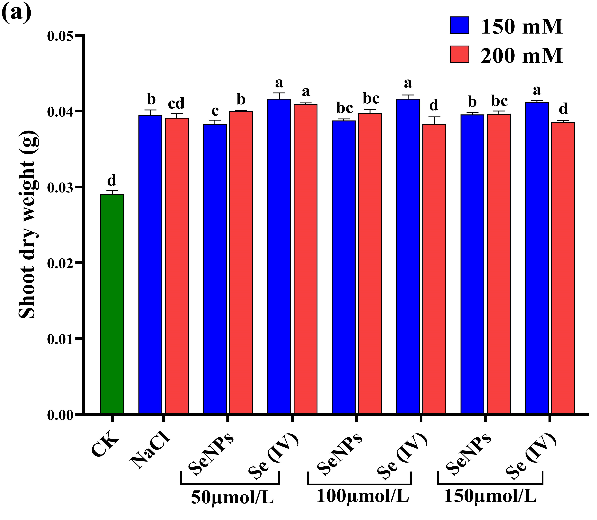 | 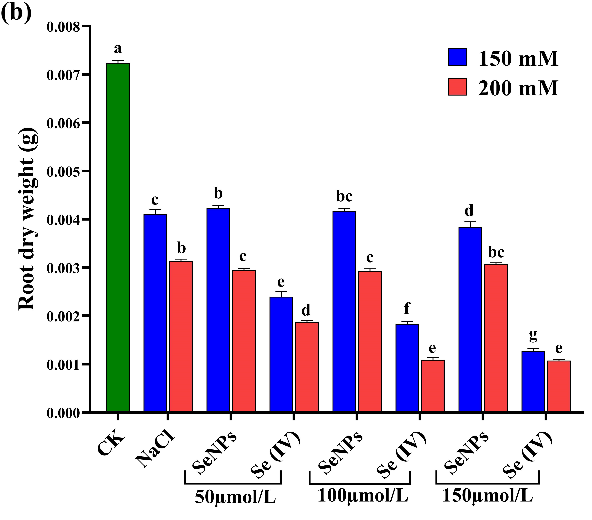 |
| --- | --- |

**Fig**. **S5**. Impacts of selenium treatments on (a) shoot dry weight (g), and (b) root dry weight (g) on Yangyou 9 under two concentrations of salt stress during the early seedling stage. Bars represent ± SE of three replicates. The different letters indicate significant differences at (*P* < 0.05) using Duncan's multiple range tests.

Shoot dry weight was increased by 36.21 and 33.98% in NaCl treatment under 150 and 200 mM, respectively. However, under 150 µmol L^-1^, bioSeNPs increased the shoot dry weight by 0.15% (150 mM) and 1.29% (200 mM), while Se (IV) improved it by 4.20% (150 mM) over the NaCl (Fig. S5a). Root dry weight was decreased by 43.52 and 58.90% in NaCl treatment under 150 and 200 mM, respectively. 150 µmol L^-1^ of Se decreased the root dry weight by 6.04 and 68.64% (150 mM), 1.93 and 65.77% (200 mM) under bioSeNPs and Se (IV), respectively versus seedling treated with NaCl alone (Fig. S5b).

**Table S1.** Sequences of primers used in this study

| **No.** | **Abbreviation** | **Gene name** | **F** | **R** |
| --- | --- | --- | --- | --- |
| **Antioxidant related genes** | | | | |
| 1 | SOD | Superoxide dismutase | ACGGTGTGACCACTGTGACT | GCACCGTGTTGTTTACCATC |
| 2 | POD | Peroxidase | ATGTTTCGTGCGTCTCTGTC | TACGAGGGTCC- GATCTTAGC |
| 3 | CAT | Catalase | TCGCCATGCTGAGAAGTATC | TCTCCAGGCTCCTTGAAGTT |
| 4 | APX | Ascorbate peroxidase | ATGAGGTTTGA CGGTGAGC | CAGCATGGGAGATGGTAGG |
| 5 | GR | Glutathione reductase | AAGCTGGAGCTGTGAAGGTT | AGACAGTGTTCGCAAAGCAG |
|  | | | | |
| **1** | E3-MUL1 | E3 ubiquitin-protein ligase MUL1 | TGCTGGCATCCTTGGCTTTGC | TGCGCTGGTCTCTGCTCGAC |
| **2** | CTP | CTP synthase (glutamine metabolic process) | TGATGAGAGACACCGGCACAGA | CCATACGACGCCCAGTCCCA |
| **3** | LBD16 | LOB domain-containing protein 16 | CGTCAGCCGCCGAAGGTACT | GCTCTGTGGCGAGACCGGAT |
| **4** | SULT1A | Aryl sulfotransferase | CCAGCTCAAGAGACTTGCGGAGT | GGCTACGCAGGGAGCAGAGC |
| **5** | SOX | Sulfite oxidase | TGGTCCTCAAGGAGACCGCAA | CCCGCCCACCTCCAGAAACC |
| **6** | Aat | Aspartate aminotransferase | AGGCCACTGCTGAGCTGCTT | AGCCGCTGCTAACCGCAGTG |
| **7** | GST-12 | glutathione S-transferase 12 | GCCTGGCCGTCCAAGCTTTC | CCATCCTCCCGGCGTCATCC |
| **8** | Trx1 | Thioredoxin 1 | GCAGACATCGACGAATGCCCTGA | GCCAAAGACGATCACGGAGCCT |
| **9** | E2 | ubiquitin-conjugating enzyme E2 | TGGTCCCAACGATGAGTCTCCTG | TCTCACACACCGGCTCACCT |
| **10** | ABCC1 | ATP-binding cassette, subfamily B (MDR/TAP), member 1 | TGCGTGGTACGGAAGCAGGT | GCCGTCCCTAGAGAAAGGCCACT |
| **11** | E3-RNF13 | E3 ubiquitin-protein ligase RNF13 | CGTCGCCGTCGCAGAGATCA | CAGGCGACGCATTGCTCGGA |
| **12** | PRt5 | 26S proteasome regulatory subunit T5 | ACAGGGAAGTGCAGCGGACTA | ACGCATCAGTGCTGGGTCCAA |
| **13** | APR | Adenylyl sulfate reductase | TTGAGGCGTGCGCTCAAGGG | CAGCTCCGCCGTCCAAACCT |
| **14** | Sulfur1;2 | Sulfate transporter 1;2 (high-affinity) | AATCGGACCCGTCGCTGTGG | ACGTGGCGGTGAAGGCAAGT |
| **15** | PLR1 | pyridoxine 4-dehydrogenase | ACCGCAAACTACGCGCCTCT | TGACTCCAACAGCTCGAACTAGCC |
| **16** | metB (CγS) | cystathionine gamma-synthase | TGTGGCCCACTGAAGGTGGT | AGGTACGCTGCGTTCGGGTT |
| **17** | metE (MET) | 5-methyltetrahydropteroyl-triglutamate-homocysteine methyltransferase | CCACCGCTATGCTCGGTGCT | CCATGGCTGGAACAGAGGCGTT |
| **18** | metC (CBL) | cystathionine beta-lyase | TGGGAATCCTACACGGGATGCAC | AGCAGCAAGAGCAGCCATTCCA |
| **19** | CYSK | cysteine synthase A | GGGAAACACTGGCCTCGGGAT | CCGCTCCTAGCGCGCGTAAA |
| **20** | SL | selenocysteine lyase | TGTGGAGGGACTGCACCCAA | GCGCAGTGGTGTCCTGACCT |
| **21** | GST-U4 | glutathione S-transferase tau 4 | TCGGCGGCAAGACAATCGGA | TTCCCAAGCCCGAGCTACGC |
| **22** | PAPSS | 3′-phosphoadenosine 5′-phosphosulfate synthase | ATCCGCGCCGGACTCATCTC | GCTTCTTCTCCCGCCGTCGT |
| **23** | CYSE | serine O-acetyltransferase | CCGACGTCGAGCAAGAACCCA | TGTTCGCCAAAGCGGAGTCCA |
| **24** | VHA-a2 | V-type H+-transporting ATPase subunit I | TGCCGTGATGCCTCTTGCAGT | TGCGGGTACCATGCCACACA |
| **25** | AtGoLS3 | galactinol synthase 3 | TCGCCGGGAACGGTGACTAC | GGTGATCCACCGGCACGTCT |
| **26** | XET | xyloglucan: xyloglucosyl transferase | GCGACGTCAACGTGGCTTGG | GGTGAGAGTAAGAAGCTGGCCGTT |
| **27** | SUlT1a | Aryl sulfotransferase | CCAGCTCAAGAGACTTGCGGAGT | GGCTACGCAGGGAGCAGAGC |
| **28** | ABCC2 | ATP-binding cassette, subfamily C (CFTR/MRP), member 2 | TGGAGTTCGGGTGGCTGTCC | ATCAACACCGGCGTGCTCCA |
| **29** | TUB | tubulin beta | CCAGCTCGAGCGCATCAACG | GGGCGGAAGGTCTGACCGTAG |
| **30** | Actin | Actin | TTGGGATGGACCAGAAGG | TCAGGAGCAATACGGAGC |
